# Induction of Breast Cancer Cell Apoptosis by TRAIL and Smac Mimetics: Involvement of RIP1 and cFLIP

**DOI:** 10.3390/cimb44100327

**Published:** 2022-10-11

**Authors:** Christian Holmgren, Ellen Sunström Thörnberg, Victoria Granqvist, Christer Larsson

**Affiliations:** Translational Cancer Research, Lund University, Medicon Village, Building 404:C3, SE-22363 Lund, Sweden

**Keywords:** Smac mimetic, TRAIL, apoptosis, breast cancer, c-FLIP, RIP1

## Abstract

Smac mimetics are a group of compounds able to facilitate cell death in cancer cells. TNF-related apoptosis-inducing ligand (TRAIL) is a death receptor ligand currently explored in combination with Smac mimetics. The molecular mechanisms determining if the combination treatment results in apoptosis are however not fully understood. In this study, we aimed to shed light on these mechanisms in breast cancer cells. Three breast cancer cell lines, MDA-MB-468, CAMA-1 and MCF-7, were used to evaluate the effects of Smac mimetic LCL-161 and TRAIL using cell death assays and Western blot. The combination treatment induces apoptosis and caspase-8 cleavage in MDA-MB-468 and CAMA-1 but not in MCF-7 cells and downregulation of caspase-8 blocked apoptosis. Downregulation, but not kinase inhibition, of receptor-interacting protein 1 (RIP1) suppressed apoptosis in CAMA-1. Apoptosis is preceded by association of RIP1 with caspase-8. Downregulating cellular FLICE-like inhibitory protein (c-FLIP) resulted in increased caspase cleavage and some induction of apoptosis by TRAIL and LCL-161 in MCF-7. In CAMA-1, c-FLIP depletion potentiated TRAIL-induced caspase cleavage and LCL-161 did not increase it further. Our results lend further support to a model where LCL-161 enables the formation of a complex including RIP1 and caspase-8 and circumvents c-FLIP-mediated inhibition of caspase activation.

## 1. Introduction

Evasion of cell death is a hallmark of cancer [1]. Overexpression of inhibitors of apoptosis proteins (IAPs) such as X-linked IAP (XIAP) and cellular IAP (cIAP) 1/2 is one mechanism that can promote cancer cell survival since they inhibit caspases and block formation of protein complexes that stimulate pro-apoptotic signaling [2].

XIAP and cIAP1/2 can be suppressed by Smac mimetics, which mimic the protein Smac, a pro-apoptotic protein that normally resides in mitochondria. Upon mitochondrial permeabilization, Smac is released and can bind and inhibit IAPs, leading to disinhibition of caspases and facilitation of pro-apoptotic signaling [3,4,5,6,7].

cIAPs also block the non-canonical nuclear factor kappa B (NF-κB) pathway by degrading NF-κB-inducing kinase (NIK). Smac mimetics can therefore induce activation of non-canonical NF-κB with subsequent transcription of NF-κB target genes such as tumor necrosis factor alpha (TNFα). Smac mimetic-induced TNFα production has been correlated to sensitivity to Smac mimetics as single agents [6,8,9]. However, most human cancer cells are resistant to Smac mimetics alone [2,10,11]. Therefore, Smac mimetics are mainly evaluated as part of combination therapies.

Activation of death receptors such as tumor necrosis factor receptor I (TNFRI) is one strategy considered in combination with Smac mimetics. This is because cIAPs can direct death receptor-signaling towards canonical NF-κB activation by ubiquitination of receptor-interacting protein 1 (RIP1) [12]. In the absence of cIAPs, the TNFR1-complex instead can activate caspase-8, resulting in activation of the extrinsic apoptotic pathway [6,8]. 

Among death receptor ligands, TNF-related apoptosis-inducing ligand (TRAIL) may be particularly promising for cancer therapy because it appears to preferentially stimulate apoptosis in tumor cells [13]. TRAIL can, similarly to TNFα, result in extrinsic apoptosis and activation of other pathways including the NF-κB pathway [14,15,16]. However, there are differences between TRAIL and TNFα signaling and the roles of cIAP1/2 and RIP1 are less established in the TRAIL pathway [17,18,19]. Smac mimetics and TRAIL in combination can induce apoptosis in cancer cells [20]. In this study, we shed light on mechanisms that may determine and mediate breast cancer cell death induced by TRAIL and Smac mimetics.

## 2. Materials and Methods

### 2.1. Cell Culture

MDA-MB-468, CAMA-1 and MCF-7 cells were from the American Type Culture Collection (ATCC, Manassas, VA, USA) and were grown in RPMI 1640 (Thermo Scientific, Waltham, MA, USA) supplemented with 10% fetal bovine serum (Biosera, Nuaille, France), 100 IU/mL penicillin, 100 µg/mL streptomycin and 1 mM sodium pyruvate (Corning, Corning, NY, USA). When indicated, cells were treated with LCL-161 (Selleckchem, Houston, TX, USA), TRAIL (Millipore, Burlington, MA, USA), TNFα (Sigma, St. Louis, MO, USA), z-VAD-fmk (Enzo Life Sciences, Farmingdale, NY, USA), necrostatin-1 (Sigma) and/or neutralizing anti-TNF antibody (Invivogen, San Diego, CA, USA, #htnfa-mab2). For siRNA transfections, 500,000 cells per a 6 cm cell culture dish were seeded. Transfections were thereafter performed as described previously [21].

### 2.2. Analysis of Cell Viability

Cells (6000 per well) were seeded in 100 µL complete medium in 96-well culture plates. After 24 h, 100 µL complete medium containing indicated compounds were added to the wells. During the final 4 h, 20 µL WST-1 (Roche, Basel, Switzerland) was added to each well. The amount of viable cells was analyzed with a WST-1 assay using a Synergy 2 Microplate Reader and Gen5 Reader Control and Data Analysis software v.1.02.8 (BioTek, Winooski, VT, USA).

### 2.3. Western Blot

Western blot was performed as described [21]. Primary antibodies used were anti-actin (1:2000 MP Biomedicals C4), anti-caspase-8 (1:300 Cell Signaling #9746 and 1:300, Abcam ab32125), anti-caspase-3 (1:1000 Cell Signaling #9662), anti-caspase-7 (1:400 Cell Signaling #12827), anti-RIP1 (1:500 BD Biosciences 610458), anti-XIAP (1:400 BD Biosciences 610762), anti-cIAP1 (1:200 R&D Systems AF8181), anti-p100/p52 (1:500 Cell Signaling #4882) and anti-c-FLIP (1:400 Cell Signaling #5634). Secondary horseradish peroxidase-labeled antibodies were from GE Healthcare and Dako and were used at 1:5000 dilutions. For the chemiluminescent reaction, Supersignal Substrate (Thermo Scientific) was used. Chemiluminescence was detected with a LAS-1000 CCD camera and Image Reader LAS-1000 Pro v2.6 software (Fujifilm, Tokyo, Japan) or an Amersham Imager 600 (GE Healthcare, Chicago, IL, USA).

### 2.4. Annexin V Analysis

Cells were seeded at a density of 2 × 10^6^ cells per 10 cm cell culture dish. After 24 h, cells were treated by changing medium to 5 mL complete medium containing indicated compounds. Cells were stained with Annexin V-APC and PI staining solution (BD Pharmingen, Franklin Lakes, NJ, USA) and were analyzed using BD FACSVerse (BD Biosciences, Franklin Lakes, NJ, USA). In addition, 10,000 events were acquired per sample and analyses were performed using BD FACSuite software v1.0.6 (BD Biosciences).

### 2.5. Flow Cytometric Analysis of DR4/DR5 Cell Surface Expression

Cell surface expression of DR4 and DR5 was performed using PE-conjugated antibodies (R&D Systems, FAB347P and FAB6311P). Cells were harvested at 70–80% confluence and were washed twice with PBS containing 1% FBS. Anti-DR4-PE or anti-DR5-PE was then added to a final concentration of 10 µg/mL to a 25 µL cell suspension containing 1 × 10^5^ cells. Control samples were incubated with isotype-matched PE-conjugated control antibodies (R&D Systems, IC002P and IC0041P). Antibody incubation was performed for 1 h at 4 °C in the dark. Cells were then washed twice with PBS containing 1% FBS and resuspended in 0.5 mL of the solution for flow cytometric analysis.

### 2.6. Analysis of BIRC3 and TNFα mRNA Levels

RNA extraction and qRT-PCR were performed as described previously [21]. Primers were from Invitrogen Life Sciences and designed using Primer Express software (Applied Biosystems; forward BIRC3: 5′-GCTGGATAACTGGAAAAGAGGA-3′, reverse BIRC3: 5′-AAGGAAAAGTAGGCTGAGAGGT-3′; forward TNFα: 5′-GCAGGTCTACTTTGGGATCATTG-3′, reverse TNFα: 5′-GCGTTTGGGAAGGTTGGA-3′).

### 2.7. Co-Immunoprecipitation

For co-immunoprecipitation, 2 × 10^6^ cells were seeded in 10 cm cell culture dishes. After 24 h, the medium was changed and the cells were pre-incubated with 20 μM z-VAD-fmk for 5 min before addition of other compounds. Cells were collected and lysed as described previously [21]. Cell lysates were incubated with caspase-8 antibodies (1:75, Cell Signaling #9746) overnight at 4 °C followed by using µMACS Protein G Microbeads and MACS Separation Columns (Miltenyi Biotec, Bergisch Gladbach, Germany).

### 2.8. Statistical Analysis

Statistical analyses were performed with IBM SPSS Statistics 22. Significance of difference in all experiments containing more than two groups was tested using analysis of variance (ANOVA) followed by Tukey’s HSD test. Significance of difference between two groups was tested using a two-tailed *t*-test.

## 3. Results

### 3.1. Effects of Smac Mimetic LCL-161 and TRAIL on Breast Cancer Cell Death

**Figure 1:**
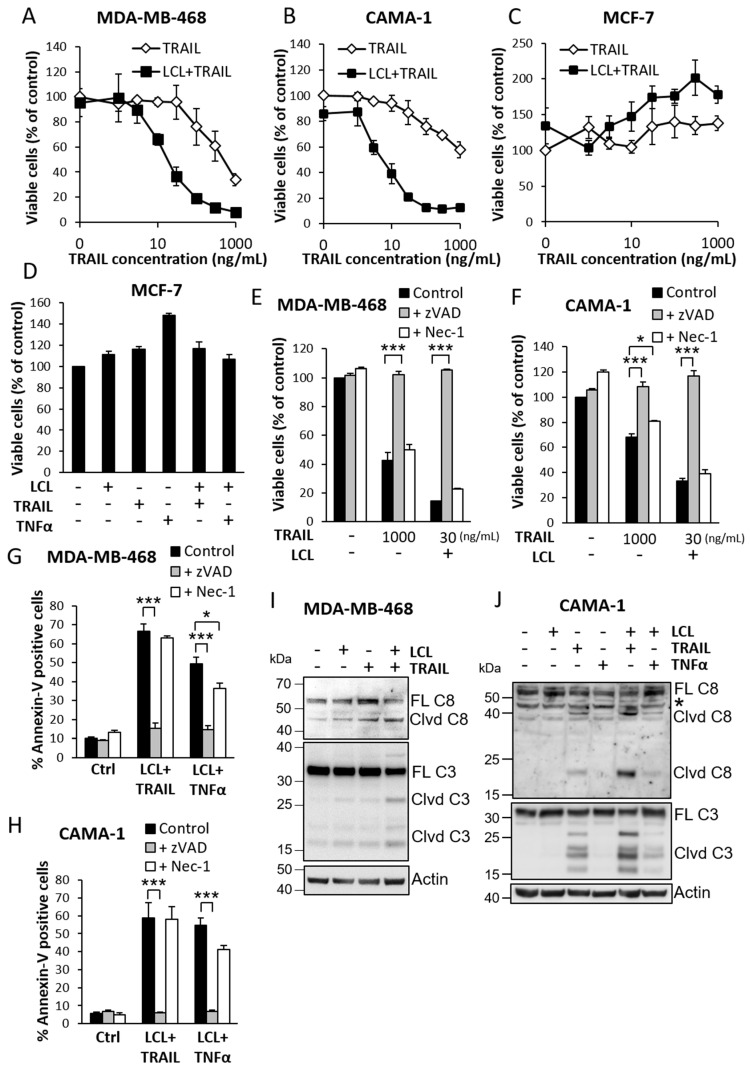
Induction of breast cancer cell death by LCL-161 and TRAIL. MDA-MB-468 cells (**A**), CAMA-1 cells (**B**) and MCF-7 cells (**C**) were treated with different concentrations of TRAIL in the presence or absence of 10 μM LCL-161 for 24 h prior to a WST-1 assay. (**D**) MCF-7 cells were treated with 10 μM LCL-161 and/or 100 ng/mL TRAIL or 10 ng/mL TNFα for 24 h prior to a WST-1 assay. MDA-MB-468 (**E**,**G**) and CAMA-1 (**F**,**H**) cells were pre-treated for 5 min with 20 μM z-VAD-fmk or 10 μM necrostatin-1 prior to treatment with 1000 ng/mL TRAIL or 10 μM LCL-161 together with 30 ng/mL TRAIL (**E**,**F**), or prior to treatment with 10 μM LCL-161 together with 30 ng/mL TRAIL or 10 ng/mL TNFα (**G**,**H**) for 24 h. Cell death was estimated with a WST-1 assay (**E**,**F**) or Annexin V assay (**G**,**H**). MDA-MB-468 (**I**) and CAMA-1 cells (**J**) were treated for 24 h (**I**) or 6 h (**J**) with 10 μM LCL-161, 100 ng/mL TRAIL and/or 10 ng/mL TNFα before analyzing caspase cleavage. Asterisk indicates a non-specific band. Data in (**A**–**H**) represent mean ± SEM from three independent experiments. * indicates *p* ≤ 0.05, *** indicates *p* ≤ 0.001. Blots are representatives of three independent experiments. Full-length blots are presented in Figure S3.

Three cell lines, resistant to Smac mimetics as single agent, were treated with combinations of the Smac mimetic LCL-161 and TRAIL. LCL-161 potentiated cell viability loss induced by TRAIL, shifting the dose–response curve to the left for both triple-negative MDA-MB-468 cells (Figure 1A) and luminal ER-positive CAMA-1 cells [22] (Figure 1B). However, neither LCL-161 nor TRAIL alone or in combination induced any decrease in viability of the luminal ER-positive MCF-7 cell line [22,23] (Figure 1C). The same result was obtained with TNFα, another death-receptor ligand (Figure 1D).

Smac mimetics may induce cell death through apoptosis and necroptosis [6,8,20,24,25]. We used the pan-caspase inhibitor z-VAD-fmk (zVAD) to inhibit the apoptotic pathway and the RIP1 inhibitor necrostatin-1 (Nec-1) to block necroptosis. In both MDA-MB-468 cells and CAMA-1 cells, zVAD treatment blocked the cell death, measured as cell viability loss and Annexin V positivity (Figure 1E–H) while Nec-1 only had minor effects. The function of Nec-1 in cultured breast cancer cell lines was validated by measuring an increase in MCF-7 cell viability through WST-1 assay (Figure S1), since it has previously been reported to increase cell proliferation in MCF-7 [26]. In addition, the LCL-161 and TRAIL combination induced cleavage of caspase-8 and caspase-3 in both cell lines (Figure 1I–J).

### 3.2. TRAIL Receptors, IAPs and Non-Canonical NF-κB

**Figure 2:**
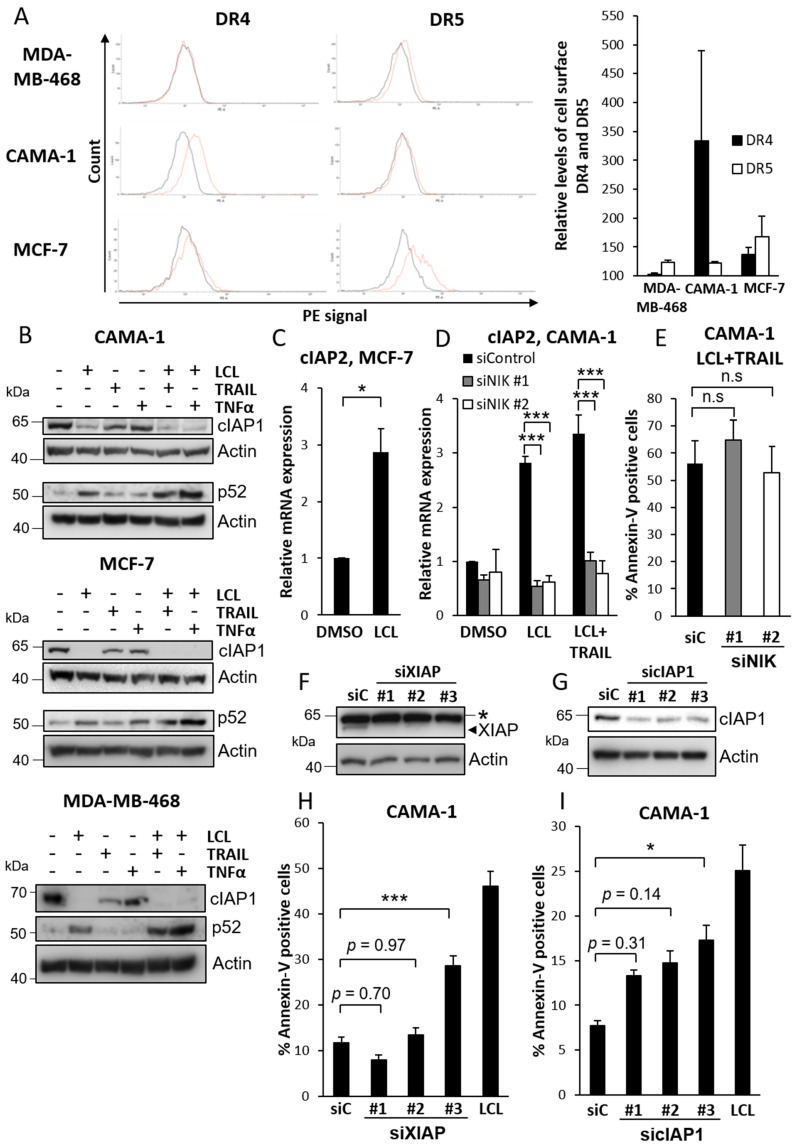
TRAIL receptors, IAPs and the effect of TRAIL and Smac mimetic. (**A**) MDA-MB-468, CAMA-1 and MCF-7 cells were stained with PE-conjugated anti-DR4 or anti-DR5 antibodies before using flow cytometry. Histograms were plotted comparing signal from each receptor (red line) with signal obtained using a corresponding PE-conjugated isotype-matched control antibody (black line). Representative histograms are shown to the left, and the results from three independent experiments were quantified and are shown to the right as percentage increase in mean fluorescence intensity compared with corresponding isotype control. (**B**) CAMA-1, MCF-7 and MDA-MB-468 cells were treated with 10 μM LCL-161, 100 ng/mL TRAIL, and/or 10 ng/mL TNFα before harvesting the cells and analyzing cIAP1 and p52 levels. MDA-MB-468 cells were treated for 6 h, whereas CAMA-1 and MCF-7 cells were treated 2 h for cIAP1 and 6 h for p52 detection. (**C**) MCF-7 cells were treated with 10 μM LCL-161 for 4 h before analyzing cIAP2 mRNA levels by qRT-PCR. (**D**,**E**) CAMA-1 cells were transfected with two different siRNA oligos targeting NIK before treating with 10 μM LCL-161 in the presence or absence of 30 ng/mL TRAIL for 4 h (**D**) or with LCL-161 and TRAIL for 24 h (**E**). cIAP2 mRNA levels and Annexin V-positive populations were thereafter analyzed by qRT-PCR (**D**) and Annexin V assay (**E**), respectively. CAMA-1 cells were transfected with three different siRNA oligos targeting XIAP for 48 h (**F**,**H**) or cIAP1 for 72 h (**G**,**I**). (**F**,**G**) Cells were harvested after transfection and knockdown efficiency was analyzed. The asterisk indicates non-specific band. (**H**,**I**) Cells were treated with 30 ng/mL TRAIL for 24 h prior to Annexin V assay. In addition, 10 μM LCL-161 were used as a positive control for cell death. Data in (**A**,**C**–**E**,**H**,**I**) represent mean ± SEM, *n* = 3. * indicates *p* ≤ 0.05, *** indicates *p* ≤ 0.001, n.s. = not significant. Blots are representatives of three independent experiments. Full-length blots are presented in Figure S3.

The lack of effect of TRAIL on MCF-7 cells could be due to the absence of receptors. We therefore analyzed with flow cytometry the presence of DR4 and DR5 on the surface of the three cell lines. We found DR5 on all cell lines, whereas DR4 seemed to be absent from MDA-MB-468 cells but present on the other two cell lines with the highest levels on CAMA-1 (Figure 2A). This is in line with another report [27] and together with our previous finding that TRAIL alone elicits interferon expression and signaling in MCF-7 cells [28] indicate that there are functional TRAIL receptors on the cell surface of MCF-7 cells.

Smac mimetic treatment generally leads to IAP downregulation followed by accumulation of NIK and activation of non-canonical NF-κB, which may contribute to cell death by induction of TNFα production [6,8]. The difference in LCL-161/TRAIL combination sensitivity between CAMA-1 and MCF-7 cells could be due to differences in these immediate effects of LCL-161. However, we found that LCL-161 induced cIAP1 downregulation in all three cell types (Figure 2B). We were not able to detect cIAP2. It has been reported that cIAP2 is not expressed in MCF-7 cells [29], which also indicate that lack of downregulation of cIAP2 is a less likely explanation to the lower effect of LCL-161. Further indicating a similar responsiveness to Smac mimetics, there was an increase in p52, an indicator of non-canonical NF-κB activation, in all three cell lines (Figure 2B). Furthermore, BIRC3/cIAP2, an NF-κB target gene, was induced by LCL-161 in both MCF-7 and CAMA-1 cells (Figure 2C,D). The induction could be blocked with siRNA targeting NIK (Figure 2D) in CAMA-1 cells indicating a mediation via non-canonical NF-κB. On the other hand, targeting NIK had no effect on cell death induced by LCL-161 and TRAIL (Figure 2E).

Smac mimetics are considered to act by inhibiting and/or downregulating XIAP and cIAP1/2. XIAP and cIAP1 were therefore individually targeted with siRNAs to assess their role. Downregulation of XIAP in CAMA-1 cells had no consistent effect on TRAIL-induced cell death compared to a control siRNA (Figure 2F,H). However, targeting cIAP1 resulted in a tendency towards an increase in TRAIL-induced cell death with one siRNA resulting in a statistically significant effect (Figure 2G,I).

### 3.3. Apoptosis Induced by LCL-161 and TRAIL Is Dependent on Caspase-8 and Independent of TNFα Induction

**Figure 3:**
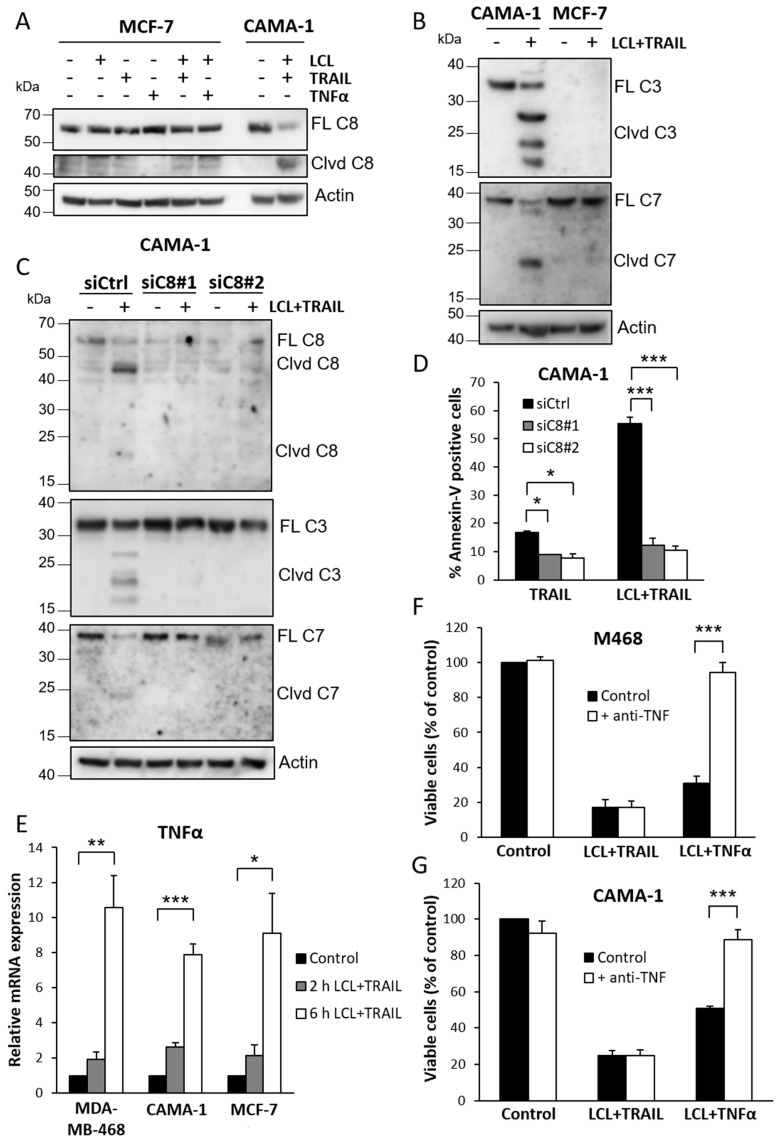
Apoptosis induced by LCL-161 and TRAIL is dependent on caspase-8 and independent of TNFα induction. (**A**,**B**) CAMA-1 and MCF-7 cells were treated for 6 h with 10 μM LCL-161, 100 ng/mL TRAIL, and/or 10 ng/mL TNFα before harvesting the cells and analyzing caspase cleavage. (**C**,**D**) CAMA-1 cells were transfected with two different siRNA oligos against caspase-8. (**C**) Cells were harvested after 6 h treatment with 10 μM LCL-161 and 100 ng/mL TRAIL following transfection. Knockdown efficiency as well as effect on downstream caspases was analyzed. (**D**) Cells were treated for 24 h with 30 ng/mL TRAIL in the presence or absence of 10 μM LCL-161 prior to Annexin V assay. (**E**) Cells were treated for indicated times with 10 μM LCL-161 and 100 ng/mL TRAIL prior to analyzing TNFα mRNA levels with qRT-PCR. (**F**,**G**) MDA-MB-468 (**F**) and CAMA-1 (**G**) cells were treated for 24 h with 10 μM LCL-161 and 30 ng/mL TRAIL or 10 ng/mL TNFα in the presence or absence of 2 µg/mL neutralizing anti-TNFα antibody prior to WST-1 assay. Data in (**D**–**G**) represent mean ± SEM from three independent experiments. * indicates *p* ≤ 0.05, ** indicates *p* ≤ 0.01, *** indicates *p* ≤ 0.001. Blots are representatives of three independent experiments. Full-length blots are presented in Figure S3.

We next analyzed if there are differences in caspase activation in CAMA-1 and MCF-7 cells. LCL-161 and TRAIL treatment at most only induced a minor caspase-8 cleavage in MCF-7 cells, contrasting what was seen for CAMA-1 cells (Figure 3A). MCF-7 cells lack expression of caspase-3 [30], but no cleavage of executioner caspase-7 was detected in MCF-7 cells, whereas caspase-7 was found to be cleaved in sensitive CAMA-1 cells (Figure 3B). Downregulation of caspase-8 in CAMA-1 cells blocked LCL-161 and TRAIL-induced cleavage of downstream caspases (Figure 3C). In addition, apoptosis induced by TRAIL alone as well as the combination treatment of LCL-161 and TRAIL was inhibited when caspase-8 was downregulated (Figure 3D).

In previous studies, TNFα induction has been described to have an important role in Smac mimetic-induced apoptosis [6,8,9]. We therefore investigated a putative role of TNFα in the apoptotic response. We found that LCL-161 and TRAIL increased TNFα mRNA levels in all three cell lines (Figure 3E). However, a neutralizing anti-TNFα antibody did not rescue the cell viability decrease induced by LCL-161 and TRAIL treatment in MDA-MB-468 or CAMA-1 cells (Figure 3F,G). The antibody completely reversed the effects of TNFα in combination with LCL-161 demonstrating its effect in this context. Thus, the apoptosis induced by LCL-161 and TRAIL occurs independently of TNFα induction.

### 3.4. LCL-161 and TRAIL Induces RIP1 Association with Caspase-8 and RIP1 Cleavage

**Figure 4:**
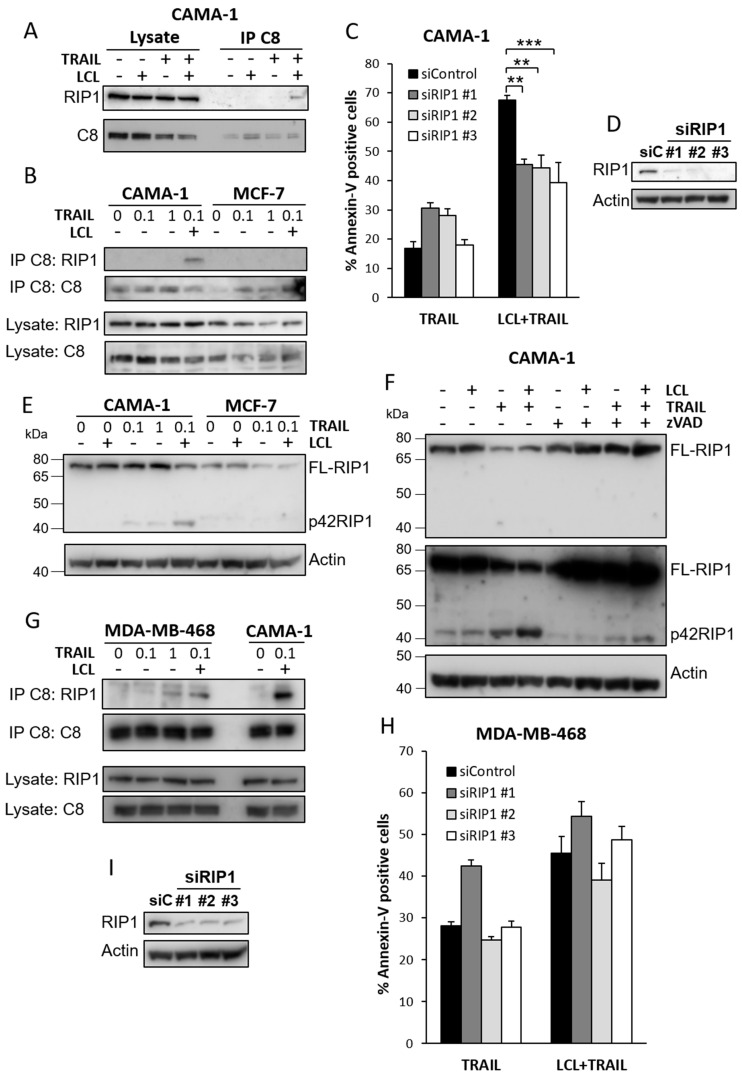
LCL-161 and TRAIL induce RIP1 association with caspase-8 and RIP1 cleavage. (**A**) CAMA-1 cells were pre-treated for 5 min with 20 μM z-VAD-fmk before treating with 10 μM LCL-161 and/or 100 ng/mL TRAIL for 2 h. Lysates were then used for caspase-8 immunoprecipitation. (**B**) CAMA-1 and MCF-7 cells were pre-treated for 5 min with 20 μM z-VAD-fmk before treating with 10 μM LCL-161 and/or 0.1 μg/mL TRAIL or 1 μg/mL TRAIL for 2 h. Lysates were then used for caspase-8 immunoprecipitation. (**C**,**D**) CAMA-1 cells were transfected with three different siRNA oligos against RIP1. (**C**) Cells were treated with 30 ng/mL TRAIL in the presence or absence of 10 μM LCL-161 for 24 h prior to Annexin V assay. (**D**) Cells were harvested after transfection and knockdown efficiency was analyzed. (**E**) CAMA-1 and MCF-7 cells were treated for 6 h with 0.1 μg/mL TRAIL or 1 μg/mL TRAIL in the presence or absence of 10 μM LCL-161. Lysates were then analyzed for RIP1 levels. (**F**) CAMA-1 cells were pre-treated for 5 min with 20 μM z-VAD-fmk before treating with 10 μM LCL-161 and/or 100 ng/mL TRAIL for 6 h. (**G**) MDA-MB-468 and CAMA-1 cells were pre-treated for 5 min with 20 μM z-VAD-fmk before treating with 10 μM LCL-161 and/or 0.1 μg/mL TRAIL or 1 μg/mL TRAIL for 2 h. Lysates were then used for caspase-8 immunoprecipitation. (**H**,**I**) MDA-MB-468 cells were transfected with three different siRNA oligos against RIP1. (**H**) Cells were treated with 30 ng/mL TRAIL in the presence or absence of 10 μM LCL-161 for 24 h prior to Annexin V assay. (**I**) Cells were harvested after transfection and knockdown efficiency was analyzed. Data in (**C**,**H**) represent mean ± SEM, *n* = 3. ** indicates *p* ≤ 0.01 and *** indicates *p* ≤ 0.001. Blots are representatives of three independent experiments. Full-length blots are presented in Figure S3.

In some cases, caspase-8 forms a cell death complex with RIP1 upon TRAIL signaling [31,32]. We wanted to analyze if this occurs in the apoptosis induced by LCL-161 and TRAIL. For this, we immunoprecipitated caspase-8 in LCL-161 and TRAIL-treated cells that had been pre-treated with zVAD in order to stabilize DISC formation [33,34,35]. RIP1 could be co-immunoprecipitated with caspase-8 from CAMA-1 cells treated with LCL-161 and TRAIL but not with either substance alone (Figure 4A). Furthermore, a high-dose TRAIL did not induce the complex in CAMA-1 cells, and it could not be observed in MCF-7 cells (Figure 4B). We next targeted RIP1 using siRNA to analyze its importance in mediating cell death. Downregulation of RIP1 in CAMA-1 cells suppressed the increase in Annexin V positivity induced by LCL-161 and TRAIL (Figure 4C,D). Furthermore, RIP1 was cleaved in CAMA-1 cells after 6 h of TRAIL treatment and LCL-161 potentiated the cleavage, generating a protein with an approximate size of 42 kDa that did not appear in MCF-7 cells under the same conditions (Figure 4E). Inhibiting caspase activity through pre-treatment with zVAD decreased the generation of this fragment (Figure 4F).

We also analyzed whether RIP1 would have a similar role in apoptosis induced by the combination of LCL-161 and TRAIL in MDA-MB-468 cells as it has in CAMA-1 cells. We found that the complex of RIP1 and caspase-8 was also induced in MDA-MB-468 cells following the combination treatment as well as single treatment with high-dose TRAIL (Figure 4G). However, the amount of RIP1 co-precipitation appeared to be lower in MDA-MB-468 than CAMA-1 cells. Suppression of RIP1 levels did not make MDA-MB-468 cells less sensitive to LCL-161 and TRAIL (Figure 4H,I).

### 3.5. c-FLIP in Apoptosis Induced by LCL-161 and TRAIL 

**Figure 5:**
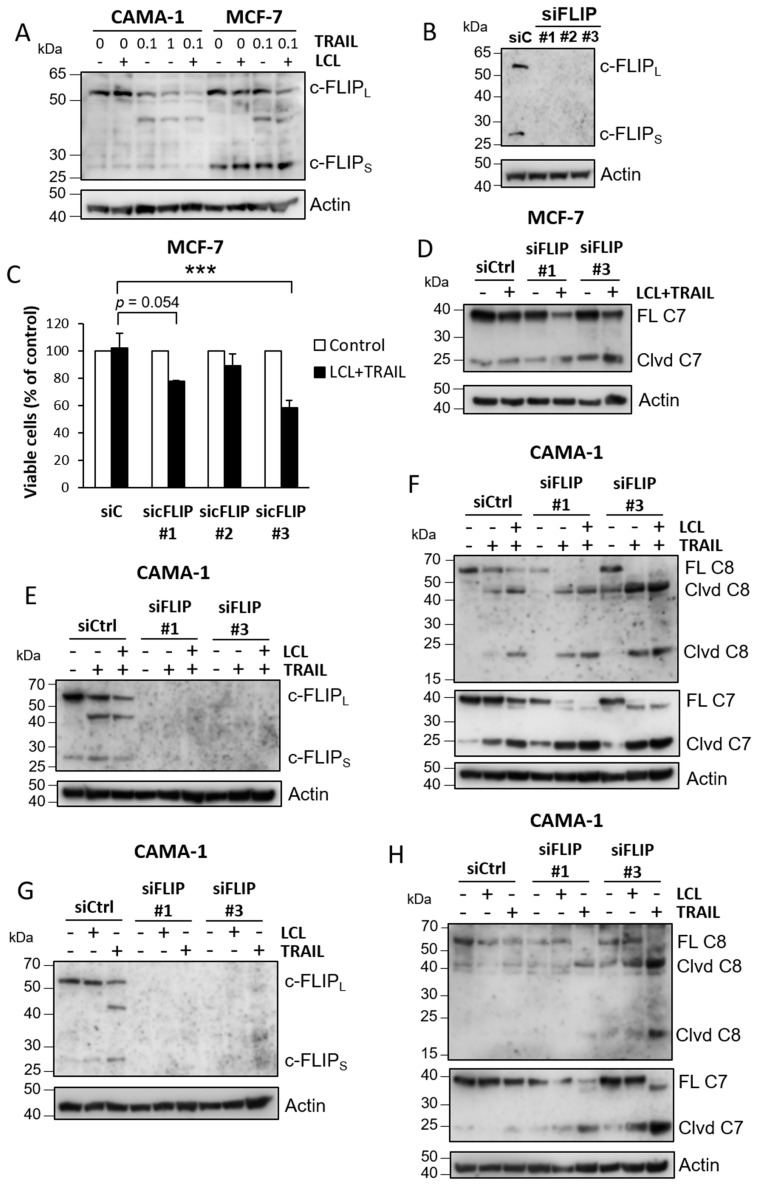
c-FLIP in apoptosis induced by LCL-161 and TRAIL. (**A**) CAMA-1 and MCF-7 cells were treated for 6 h with 10 μM LCL-161 and/or 0.1 μg/mL TRAIL or 1 μg/mL TRAIL. Lysates were then analyzed for c-FLIP levels. (**B**,**C**) MCF-7 cells were transfected with three different siRNA oligos targeting c-FLIP. (**B**) Cells were harvested after transfection and knockdown efficiency were analyzed. (**C**) MCF-7 cells were re-seeded into 96-well plates after transfection followed by treatment with 10 μM LCL-161 and 100 ng/mL TRAIL for 24 h prior to a WST-1 assay. (**D**) MCF-7 cells were transfected with two different siRNA oligos against c-FLIP before 6 h treatment with 10 μM LCL-161 and 100 ng/mL TRAIL and analysis of caspase-7 levels. (**E**–**H**) CAMA-1 cells were transfected with two different siRNA oligos against c-FLIP before 6 h treatment with 10 μM LCL-161 and/or 100 ng/mL TRAIL as indicated. Knockdown efficiency (**E**,**G**) and caspase cleavage (**F**,**H**) were analyzed by Western blot. Data in (**C**) represent mean ± SEM, *n* = 3. *** indicates *p* ≤ 0.001. Blots are representatives of three independent experiments. Full-length blots are presented in Figure S3.

We observed differences between CAMA-1 and MCF-7 cells in cellular FLICE-like inhibitory protein (c-FLIP) levels (Figure 5A). Compared to CAMA-1, MCF-7 cells had higher levels of the short isoform c-FLIP_S_, which is known to inhibit caspase-8 activation, but similar levels of the longer variant (c-FLIP_L_), which can either potentiate or suppress caspase-8 activity depending on the stoichiometric relation between them [36,37,38]. In CAMA-1, c-FLIP_L_ levels were decreased by TRAIL stimulation, an effect that was enhanced by LCL-161. We then investigated if MCF-7 sensitivity to LCL-161 and TRAIL could be potentiated by decreasing c-FLIP levels. Downregulation showed a tendency towards increased sensitivity to LCL-161 and TRAIL in terms of cell viability with one siRNA resulting in a statistically significant effect (Figure 5B,C). The effect correlated with increased caspase-7 cleavage after LCL-161 and TRAIL treatment in the absence of c-FLIP (Figure 5D). 

In CAMA-1 cells, downregulation of c-FLIP potentiated TRAIL-induced cleavage of caspase-8 and caspase-7 to the extent that addition of LCL-161 barely had any further potentiating effect (Figure 5E,F). Downregulation of c-FLIP also had a slight potentiating effect on caspase cleavage induced by LCL-161 alone (Figure 5G,H).

**Figure 6:**
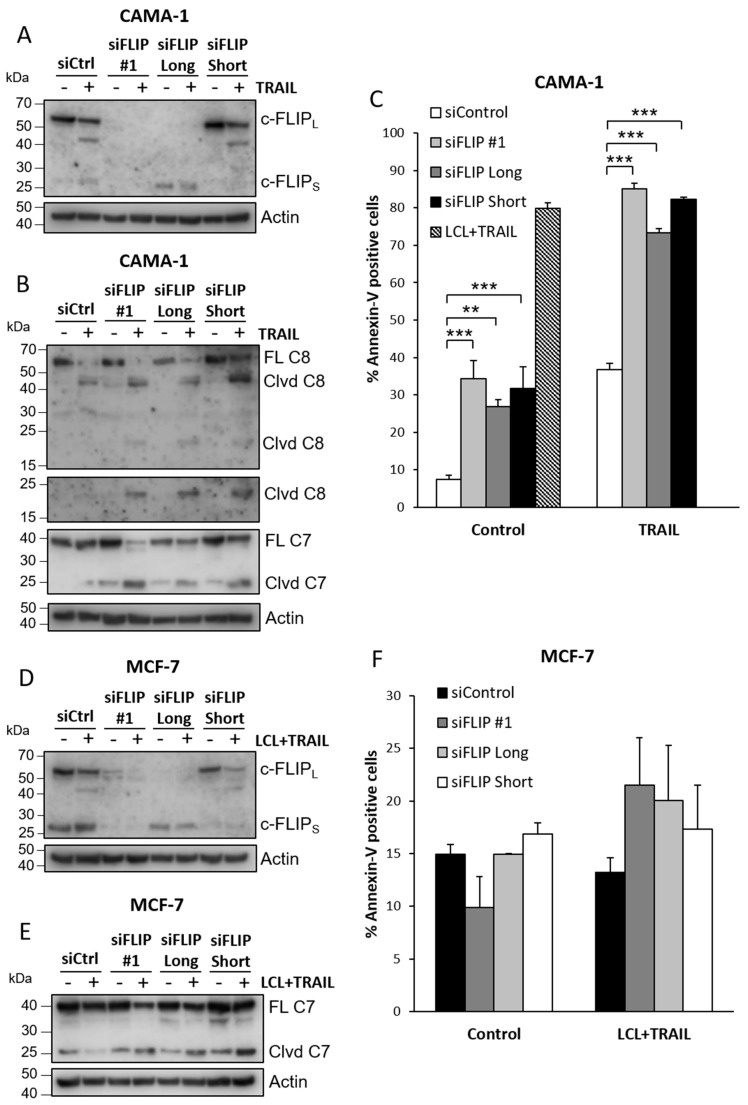
Roles of c-FLIP isoforms in apoptosis induced by LCL-161 and TRAIL. CAMA-1 (**A**–**C**) or MCF-7 cells (**D**–**F**) were transfected with siRNA targeting both the long and short c-FLIP isoforms (siFLIP #1) or with siRNA oligos targeting specific isoforms before 6 h (**A**,**B**,**D**,**E**) or 24 h (**C**,**F**) treatment with 100 ng/mL TRAIL or with 10 μM LCL-161 and 100 ng/mL TRAIL as indicated. Knockdown efficiency (**A**,**D**) and caspase cleavage (**B**,**E**) were analyzed by Western blot, and cell death was analyzed with an Annexin V assay (**C**,**F**). Data in (**C**,**F**) represent mean ± SEM, *n* = 3. ** indicates *p* ≤ 0.01, *** indicates *p* ≤ 0.001. Blots are representatives of three independent experiments. Full-length blots are presented in Figure S3.

Since c-FLIP isoforms have been described to have opposing effects on caspase-8 activation in certain contexts [37,39], we selectively downregulated the isoforms and compared the effects on LCL-161- and TRAIL-induced caspase cleavage. In both MCF-7 and CAMA-1 cells, downregulation of individual c-FLIP isoforms gave similar effects on LCL-161 and TRAIL-induced caspase cleavage as downregulation of both isoforms (Figure 6A,B,D,E). We also analyzed induction of cell death (Figure 6C,F). For CAMA-1 cells, downregulation of either isoform resulted in an increase in Annexin-V positivity and following TRAIL-stimulation the magnitude of the effect replicated what was observed for TRAIL stimulation in the presence of LCL-161 (Figure 6C). For MCF-7, the effect of downregulation was not as evident, and no significant effect was found (Figure 6F). However, for the difference in the number of Annexin-V-positive cells between cells treated with TRAIL and LCL-161 with cells that were not treated, there was a significant effect when comparing siFLIP #1 with siControl (mean +/− SEM: −1.70 +/− 1.11 for siControl and 11.57 +/− 3.19 for siFLIP#1, *p*-value = 0.041 with a two-tailed *t*-test). This was not seen for siRNA targeting c-FLIP_L_ and c-FLIP_S_.

## 4. Discussion

In this study, we found that LCL-161 potentiates TRAIL-induced apoptosis in the basal-like MDA-MB-468 and the luminal CAMA-1 cell lines but not in luminal MCF-7 cells. A research map summarizing this study and the findings can be found in Figure S2.

The most widely accepted model today stipulates that TRAIL stimulation results in recruitment of FAS-associated death domain protein (FADD) and caspase-8 to the TRAIL receptor, which is followed by cleavage and activation of caspase-8. In addition, a secondary complex can be formed, comprised of several proteins including RIP1, TRAF2, FADD, and caspase-8, which can activate signaling pathways such as the NF-κB pathway [17,18,33]. Here, cIAP1 and cIAP2 direct TNFα signaling towards activation of the canonical NF-κB through ubiquitination of RIP1 [12]. However, the role of cIAP1/2 is not completely clear in a TRAIL context but reports indicate an anti-apoptotic effect [40,41,42], which potentially could function in a similar manner as in TNFα signaling through RIP1 ubiquitination. We found that cIAP1 downregulation tended to increase susceptibility to TRAIL in CAMA-1 cells, suggesting that degradation of cIAP1 is an important step in the LCL-161 potentiation of TRAIL and that cIAP1, in line with other cell types, have an anti-apoptotic role in TRAIL signaling also in breast cancer cells. The limited effect of the siRNA could be due to incomplete downregulation. Other studies have found that XIAP can be a regulator of TRAIL sensitivity [43,44]. However, our results showed that downregulation of XIAP did not increase TRAIL-induced cell death.

Apoptosis induction by Smac mimetics as single agents has in general been found to be mediated via induction of TNFα and further autocrine/paracrine TNFα signaling [6,8,9]. TNFα expression was found to be induced by the combination of LCL-161 and TRAIL in all cell lines investigated. However, a TNFα-blocking antibody did not affect cell death induction by LCL-161 and TRAIL conceivably, discarding the hypothesis of a role for TNFα in the effect.

Several studies have linked cell death induced by Smac mimetics and TRAIL to formation of a complex containing RIP1, FADD and caspase-8 [31,32,45]. These components can constitute a death-inducing complex called the ripoptosome [39,46,47]. In our study, RIP1, the key component of these complexes, associated with caspase-8 in CAMA-1 cells only when LCL-161 and TRAIL were used in combination. The complex was also found after treatment in MDA-MB-468 cells but not in MCF-7 cells. It is possible that the lack of complex formation in MCF-7 cells could be an explanation to the lack of caspase cleavage and apoptosis in this cell line. Downregulation of RIP1 using a siRNA approach reduced apoptosis induced by the combination treatment in CAMA-1 cells. In contrast, the RIP1 kinase inhibitor necrostatin-1 had no effect. In MDA-MB-468 cells, siRNA-mediated downregulation of RIP1 did not influence cell death induced by TRAIL and LCL-161, despite a complex formation. The reason for the different effects of RIP1 knockdown is not clear. It could be related to a lower amount of complex formed in MDA-MB-468 cells. Another possible explanation may be that apoptosis induced by LCL-161 and TRAIL is mediated at least partly through RIP1 in CAMA-1 cells, whereas it is mediated through an RIP1-independent pathway in MDA-MB-468 cells. However, there also seemed to be less efficient RIP1 downregulation in MDA-MB-468 cells which may complicate the interpretation.

There are discrepant results regarding the importance of kinase activity in RIP1-dependent apoptosis [32,39,45,46]. Our data suggest a kinase-independent role for RIP1 for optimal apoptosis induction in CAMA-1 cells by LCL-161 and TRAIL. We also observed that RIP1 was cleaved into a ~42 kDa fragment lacking the kinase domain upon exposure to LCL-161 and TRAIL [48]. This cleavage has been described to be mediated by caspase-8 [48], which is in line with our findings where the cleavage could be inhibited with z-VAD-fmk. The fragment generated has been shown to promote cell death [48,49,50]. 

Another potential regulator of caspase-8 activation is c-FLIP, a catalytically inactive caspase-8 homologue, which has been implicated in TRAIL resistance in HER2-positive breast cancer cells [51] and to be downregulated in association with increased TRAIL sensitivity during endocrine resistance [52]. c-FLIP was downregulated during apoptosis in CAMA-1 cells but less affected by the same treatment in MCF-7 cells. Furthermore, basal levels of c-FLIP_S_, the isoform most strongly associated with caspase-8 inhibition [53,54], were higher in MCF-7 than CAMA-1 cells. We also found that downregulation of c-FLIP resulted in slight increases in TRAIL- and LCL-161-mediated cell death and caspase-7 activation in MCF-7 cells. A potentiation of caspase activation and cell death following c-FLIP downregulation was also observed in CAMA-1 cells for TRAIL treatment alone. In fact, if c-FLIP or either of the long or short isoform is downregulated, LCL-161 barely has any further potentiating effect on TRAIL-mediated caspase-8 and caspase-7 cleavage. Thus, LCL-161 overcomes the apoptosis inhibition by c-FLIP in CAMA-1 cells. In contrast to c-FLIP_S_, c-FLIP_L_ can either promote or inhibit caspase-8 activation depending on cellular context [55,56]. However, specific downregulation of individual c-FLIP isoforms did not result in any marked differences in caspase activation or cell death induction compared to downregulation of both isoforms in this study, suggesting that both isoforms negatively regulate caspase activation and apoptosis induced by LCL-161 and TRAIL under these circumstances.

## 5. Conclusions

Smac mimetic LCL-161 can increase TRAIL sensitivity in luminal CAMA-1 and triple-negative MDA-MB-468 but not in luminal MCF-7 cells. In CAMA-1 cells, the same effect can be obtained by downregulating c-FLIP and a kinase-independent RIP1 function may be of importance for optimal apoptosis induction. The resistance in MCF-7 cells is conceivably upstream of caspase-8 cleavage and can be partially reversed by targeting c-FLIP.

## Data Availability

All data generated or analyzed during the current study are included in this published article.

## Supplementary Materials

The following supporting information can be downloaded at: https://www.mdpi.com/article/10.3390/cimb44100327/s1, Figure S1: Nec-1 validation: MCF-7 cells were treated for 24 h with 10 μM necrostatin-1 (Nec-1) prior to WST-1 assay. Data represent mean ± SEM, *n* = 3. ** indicates *p* ≤ 0.01. Figure S2: Research map: Research map summarizing the research questions, routes and findings. Figure S3: Full-length blots: Full-length versions of blots shown in manuscript figures.
