# Supplementary material for: Induction of Breast Cancer Cell Apoptosis by TRAIL and Smac Mimetics: Involvement of RIP1 and cFLIP
Source: Curr Issues Mol Biol. 2022 Oct 11;44(10):4803–21. doi: 10.3390/cimb44100327 (PMC9600666; doi:10.3390/cimb44100327)

Full-length blots from Figure 1

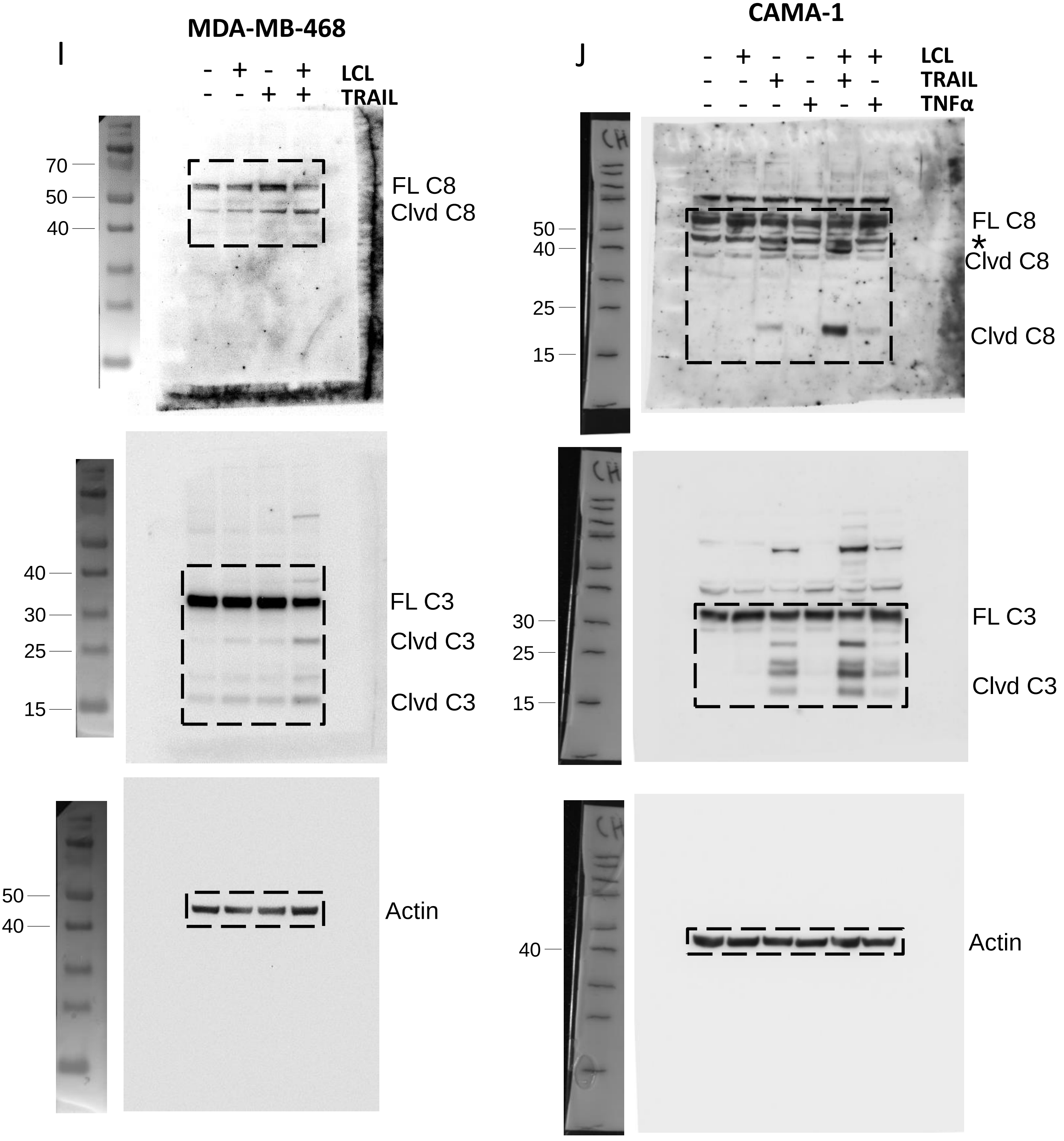

Full-length blots from Figure 2

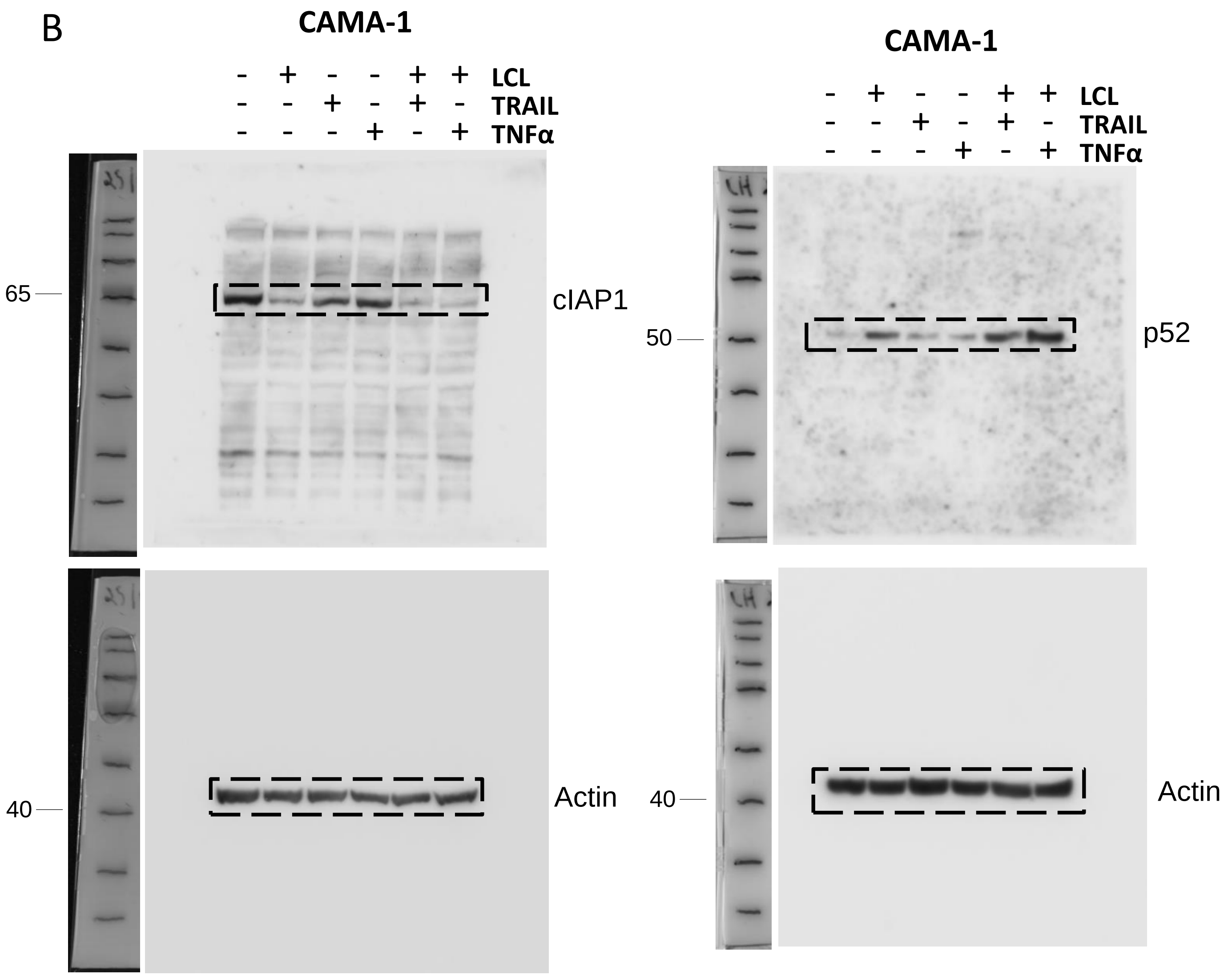

Full-length blots from Figure 2 continued

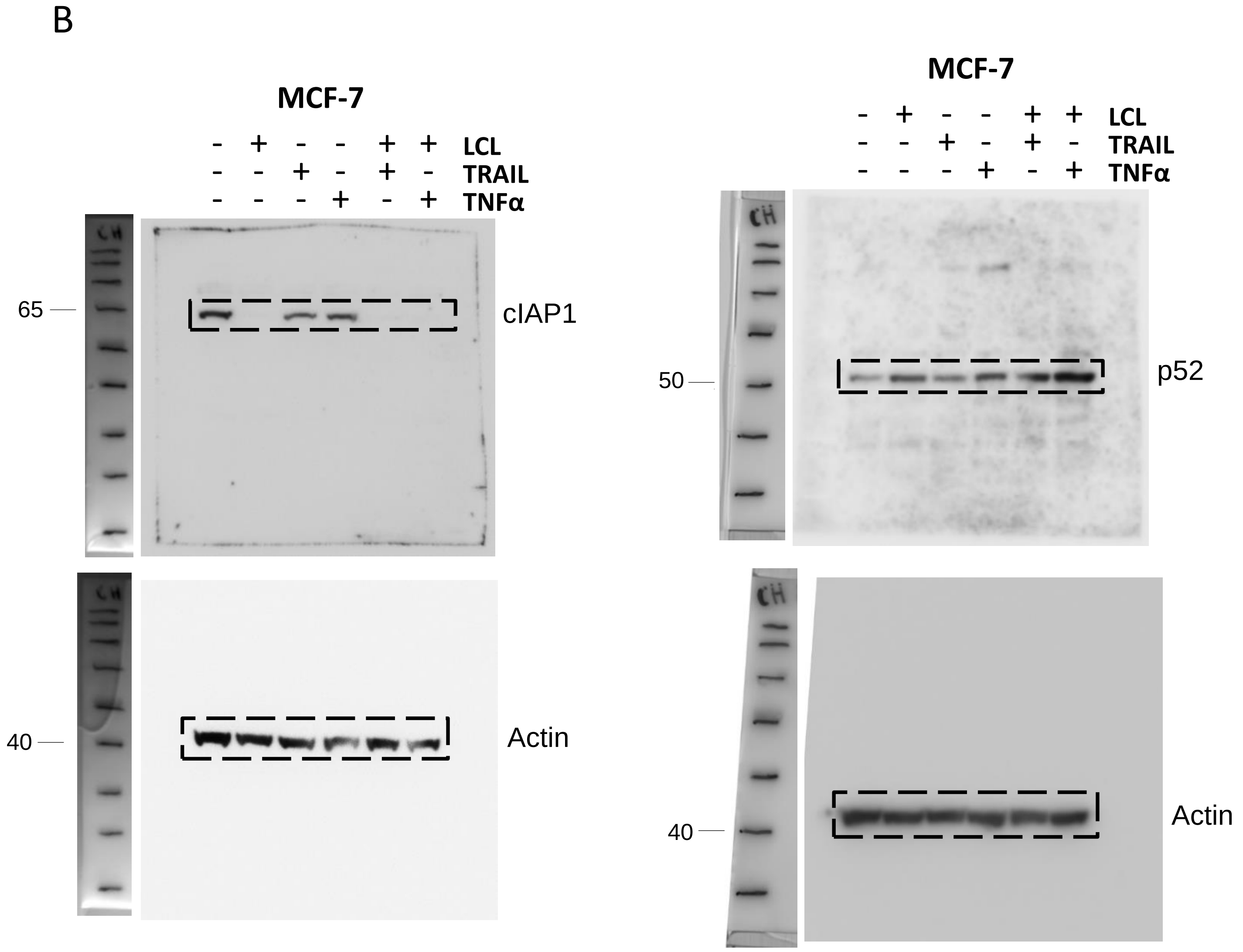

Full-length blots from Figure 2 continued

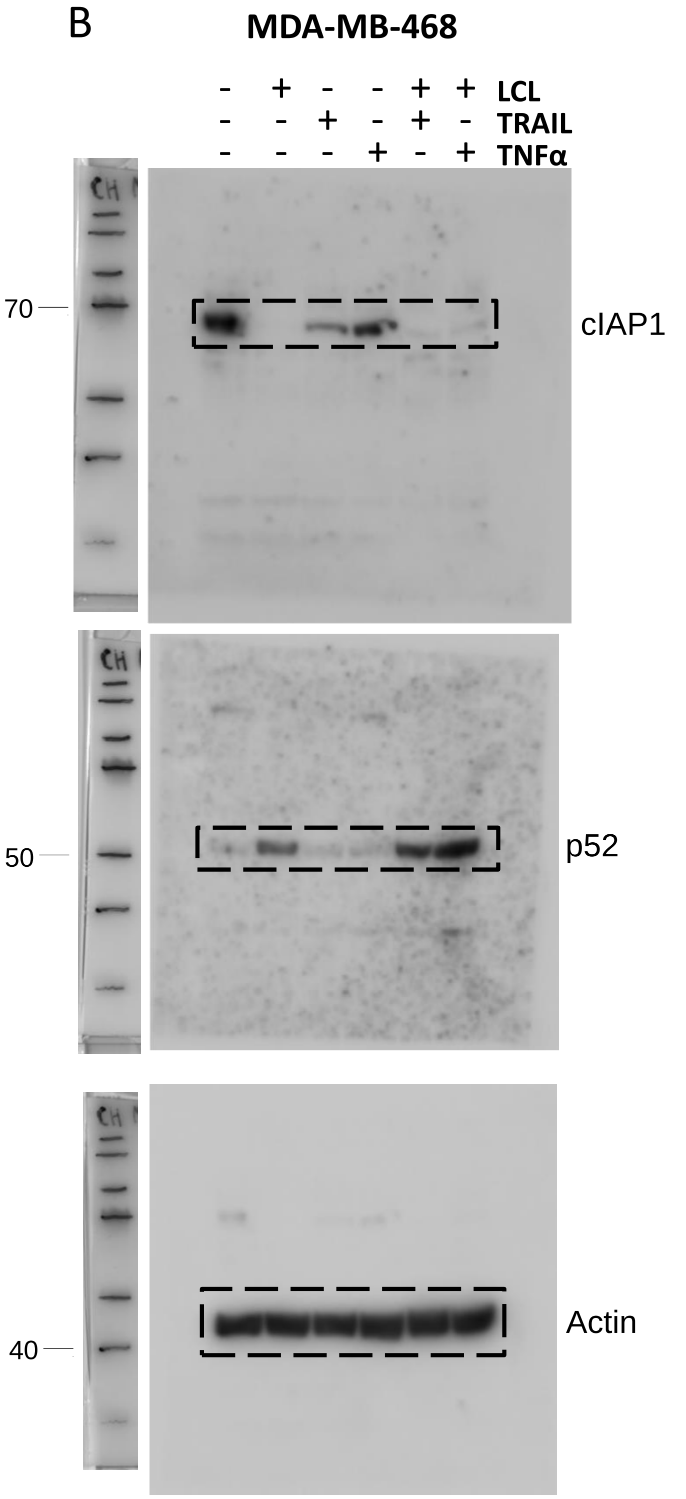

Full-length blots from Figure 2 continued

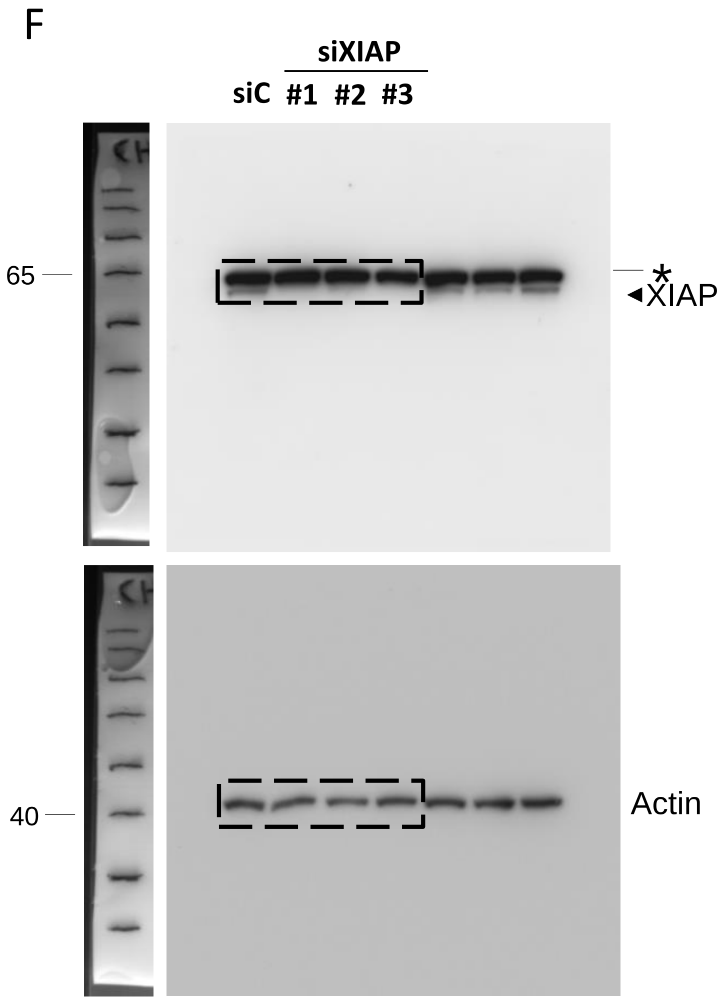

Bands in lanes outside the cropped area are from another experiment

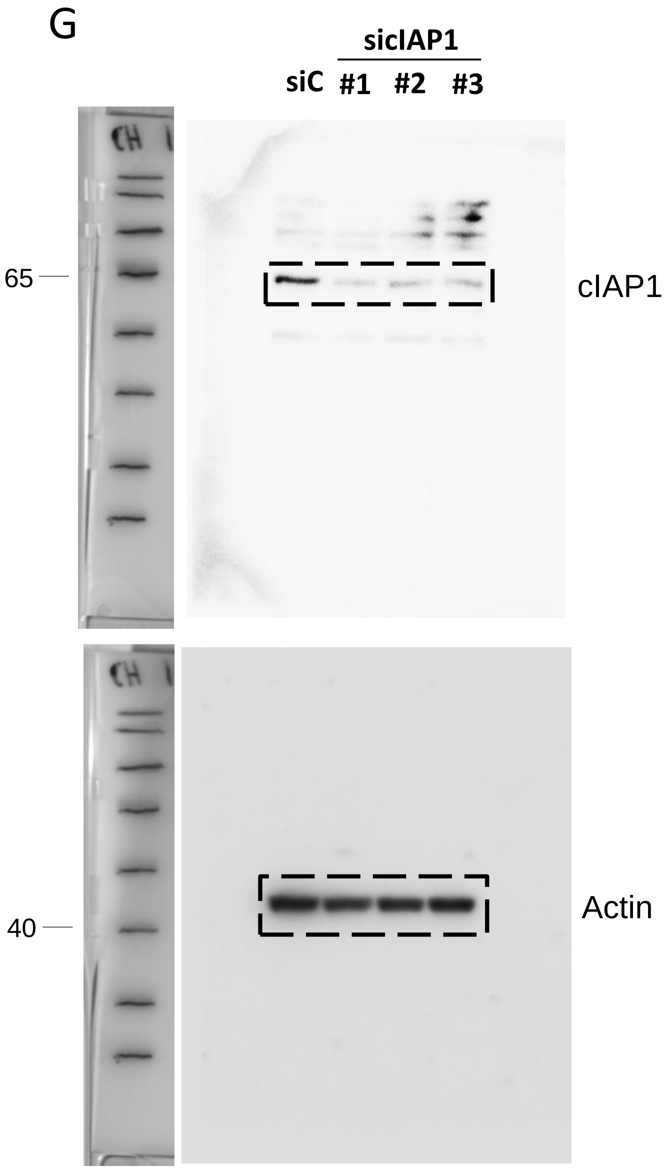

Full-length blots from Figure 3

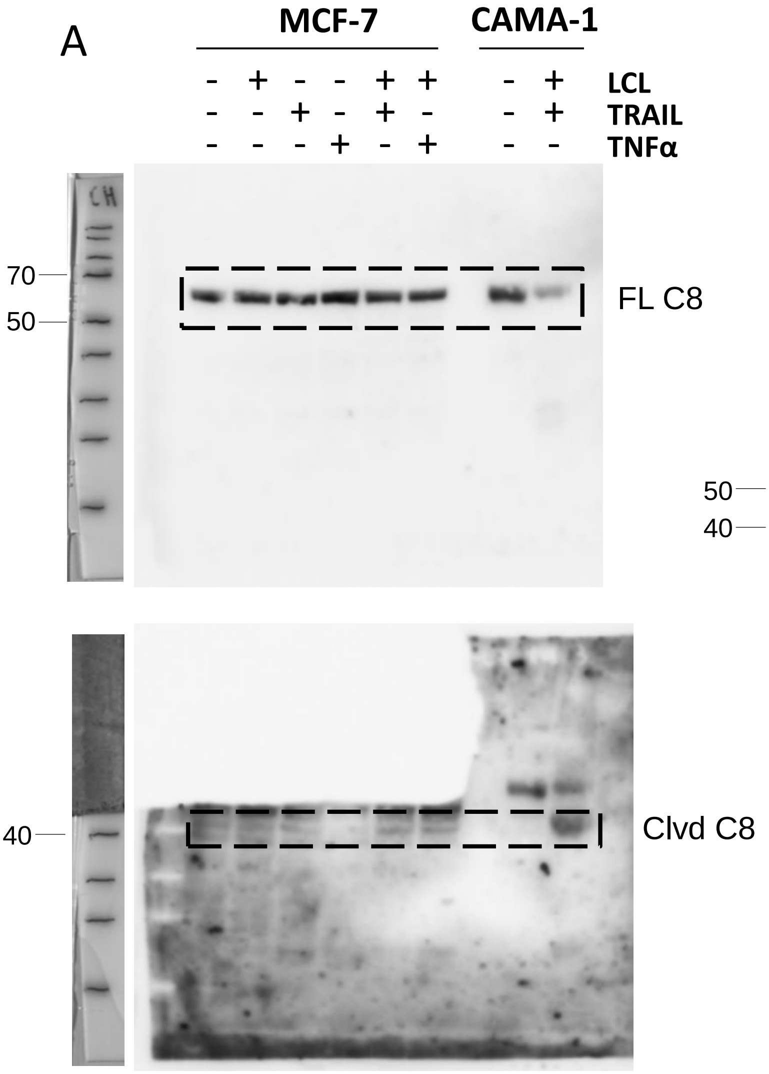

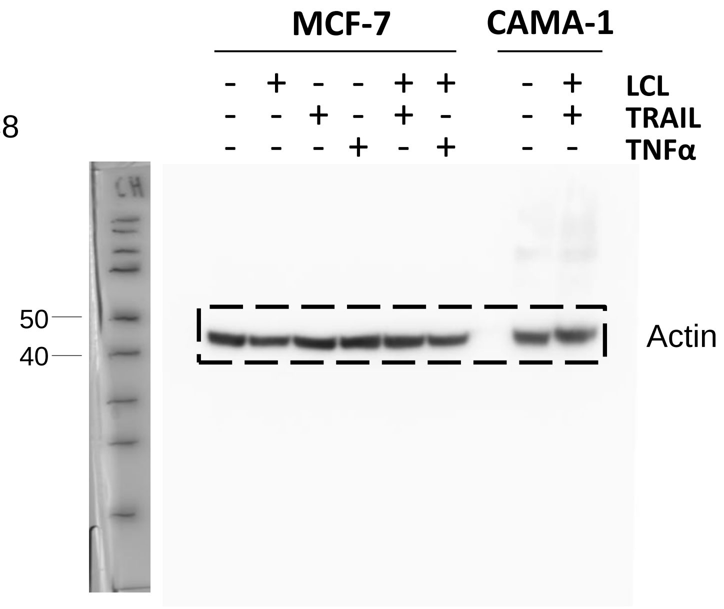

Part of the membrane was blocked to allow better visibility of cleaved caspase-8. Below is an image of the full-length blot

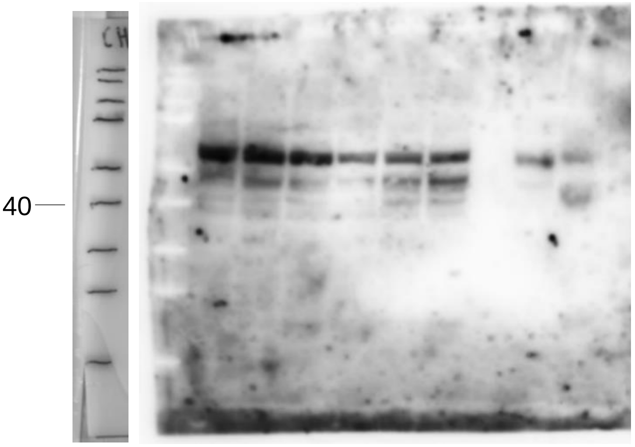

Full-length blots from Figure 3 continued

B

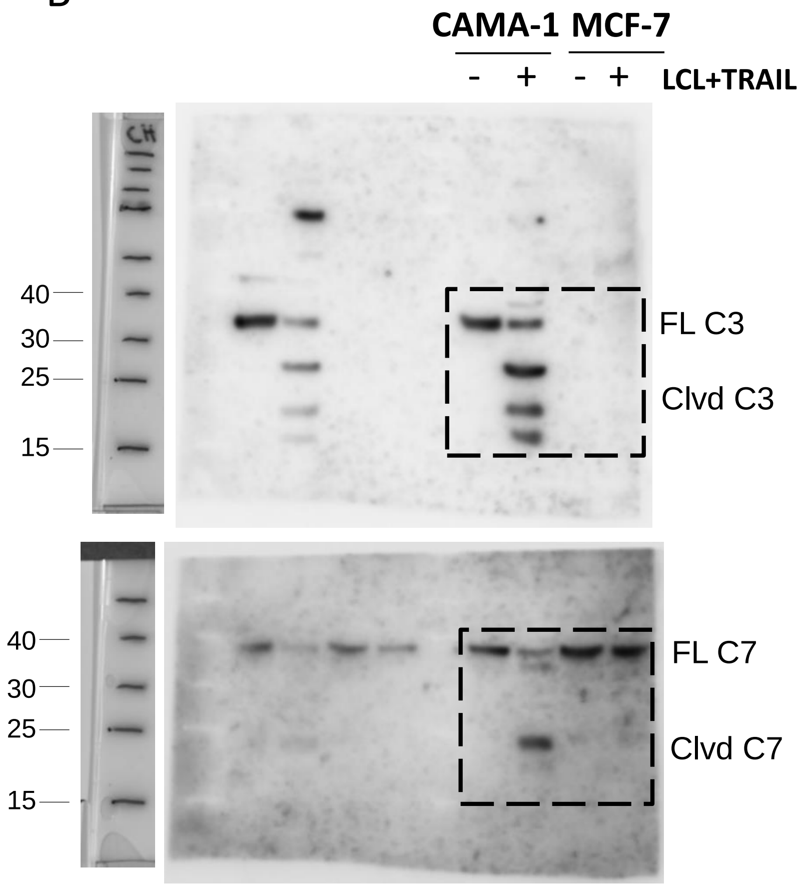

Bands in lanes outside the cropped area are from another experiment

Part of the membrane was blocked to allow better visibility of cleaved caspase-7. Below is an image of the full-length blot

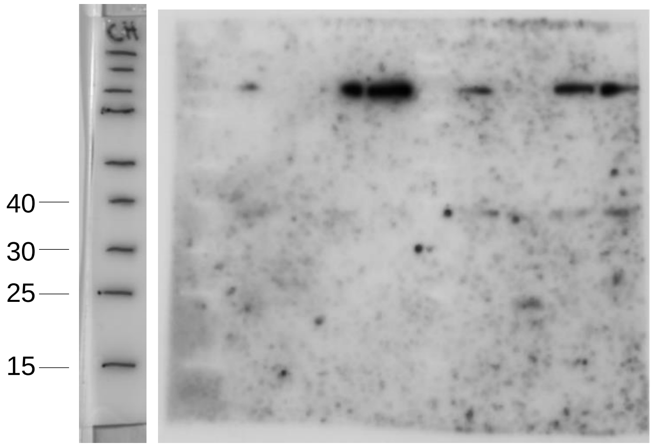

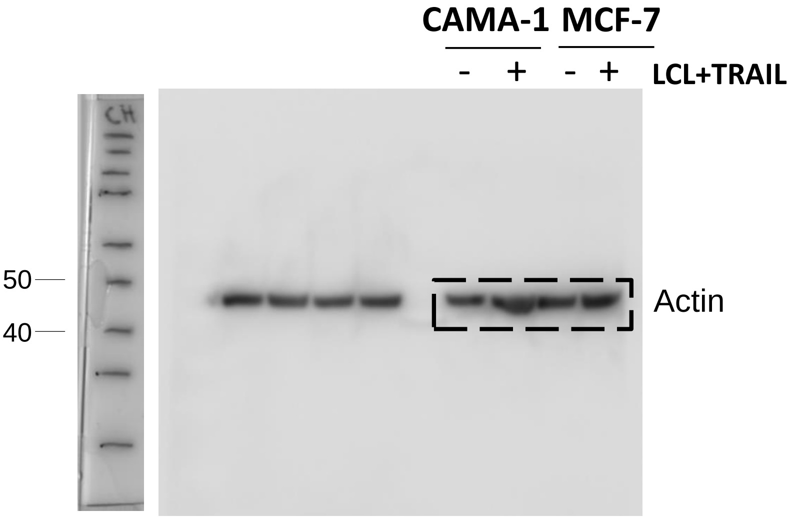

Full-length blots from Figure 3 continued

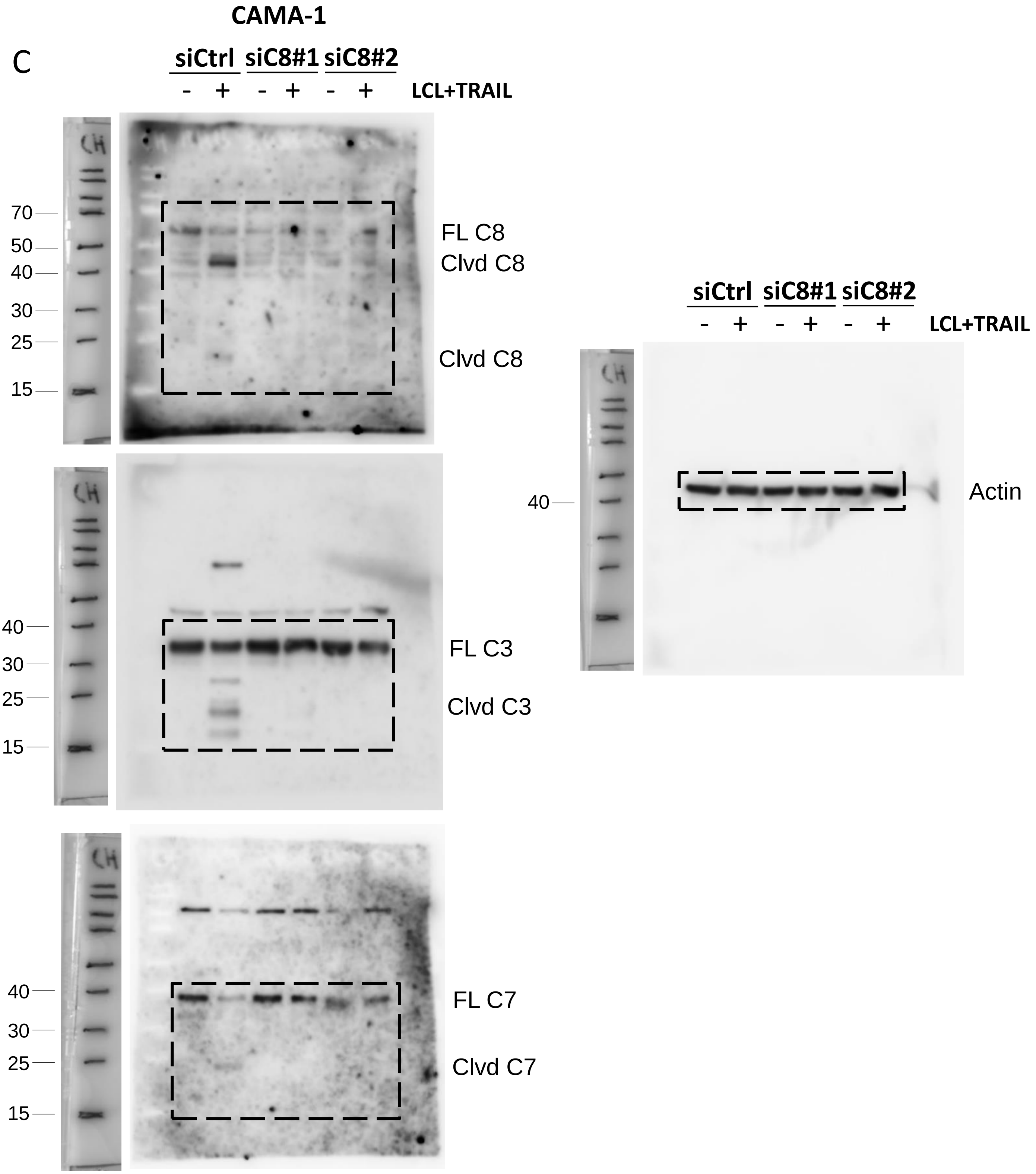

Full-length blots from Figure 4

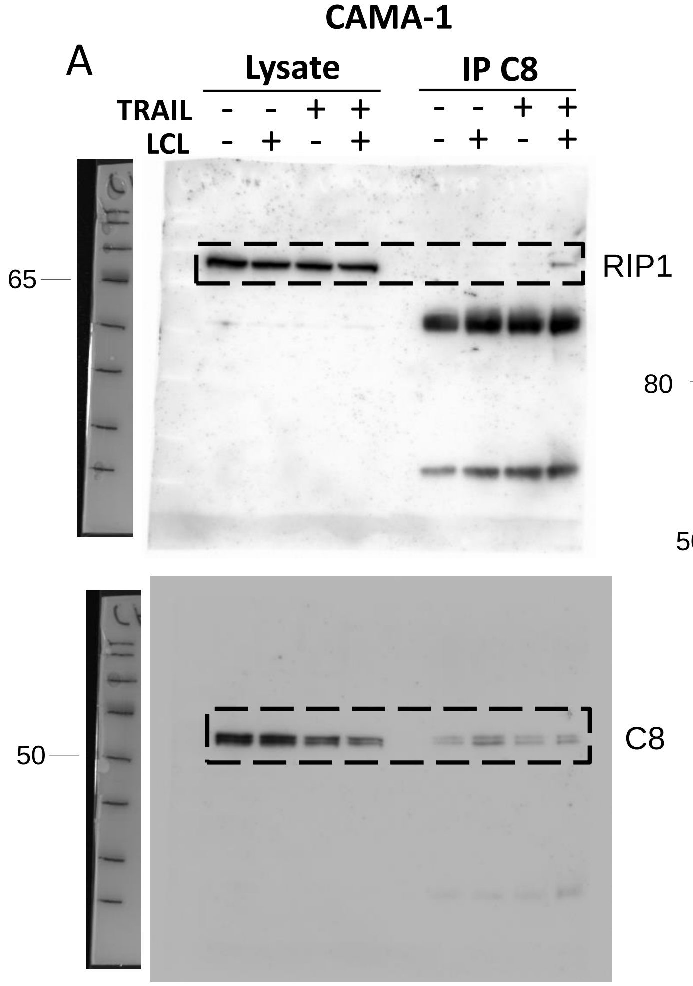

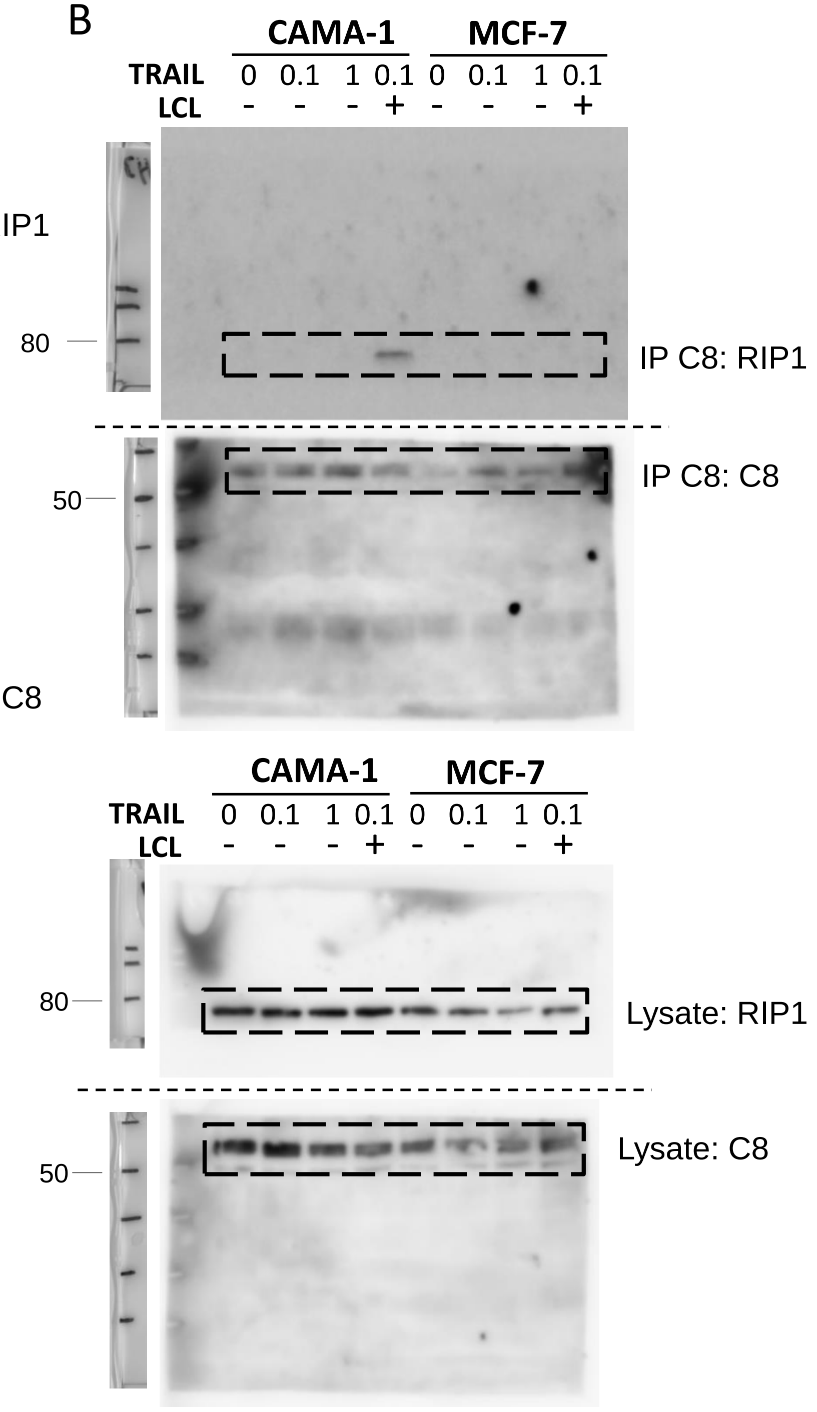

The membranes were cut around the 65 kDa mark after transfer, Caspase-8 and RIP1 were blotted on the same membrane

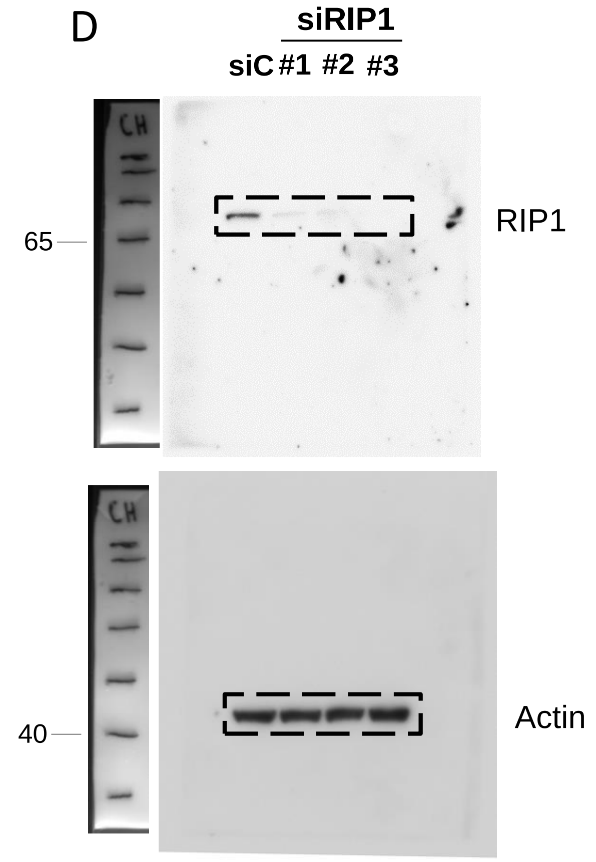

Full-length blots from Figure 4 continued

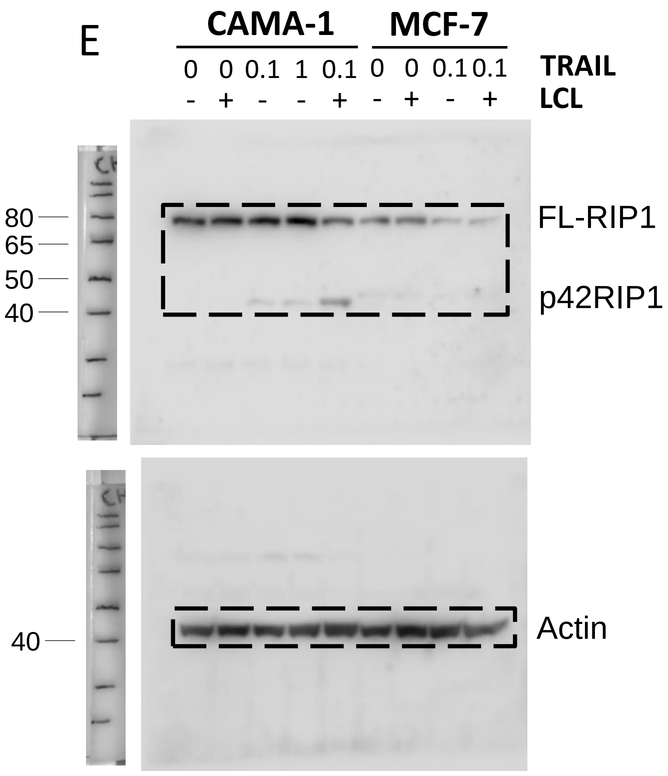

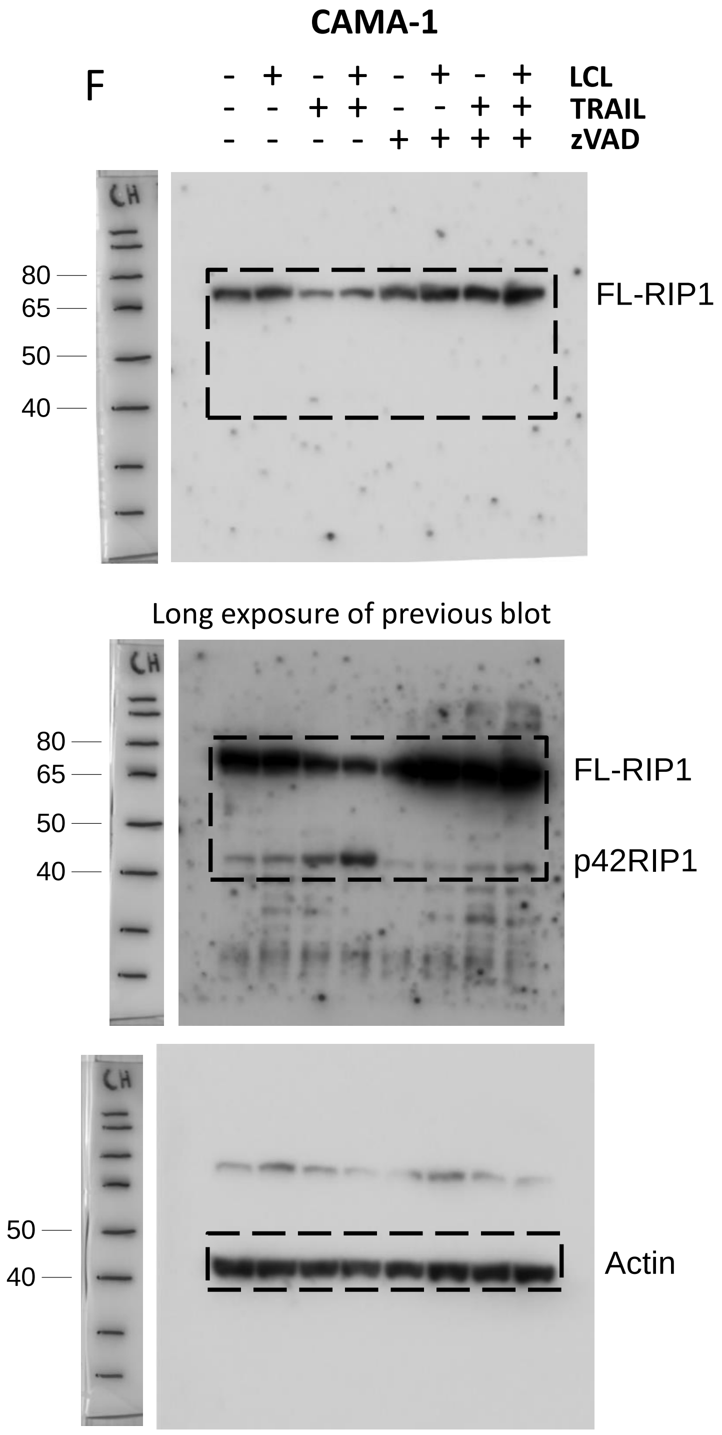

Full-length blots from Figure 4 continued

G

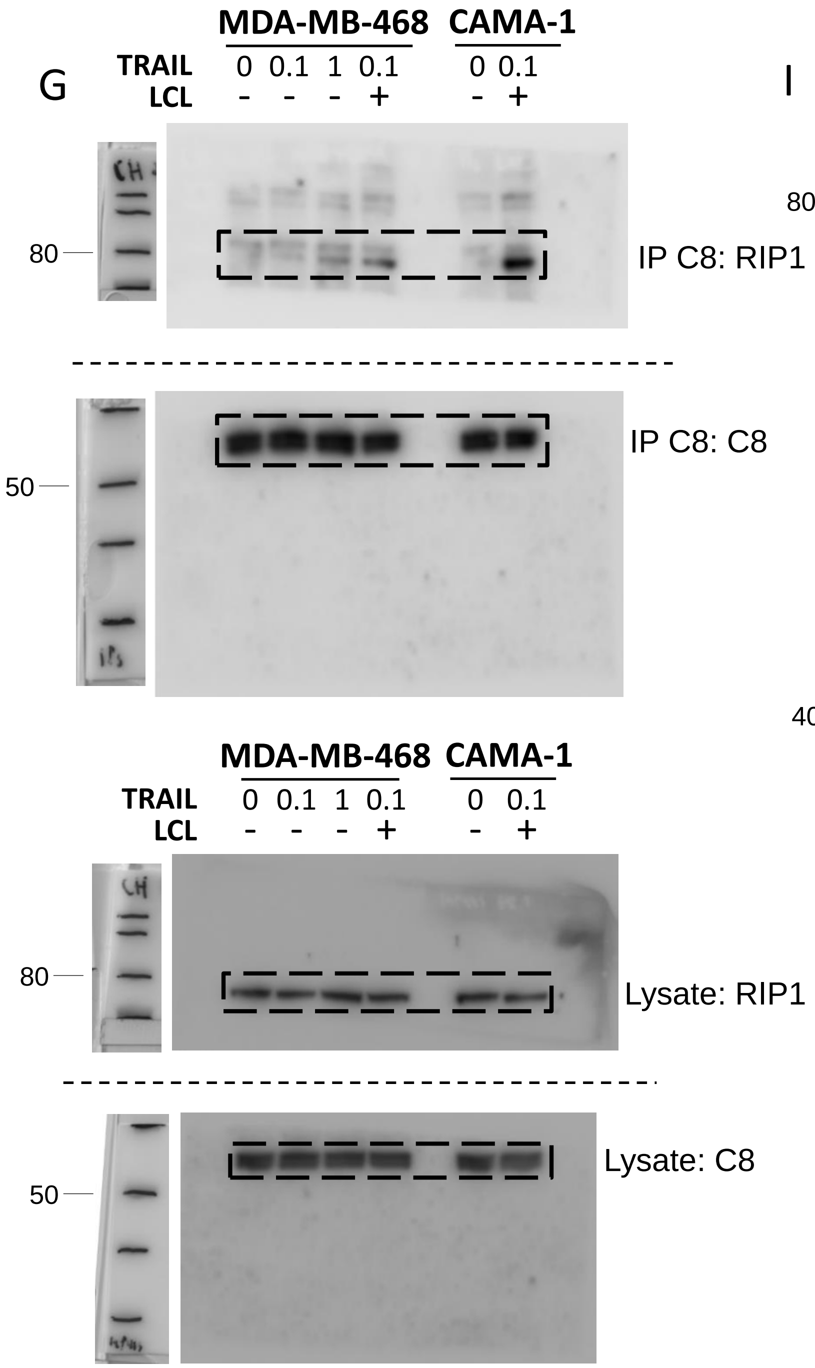

The membranes were cut around the 65 kDa mark after transfer, Caspase-8 and RIP1 were blotted on the same membrane

I

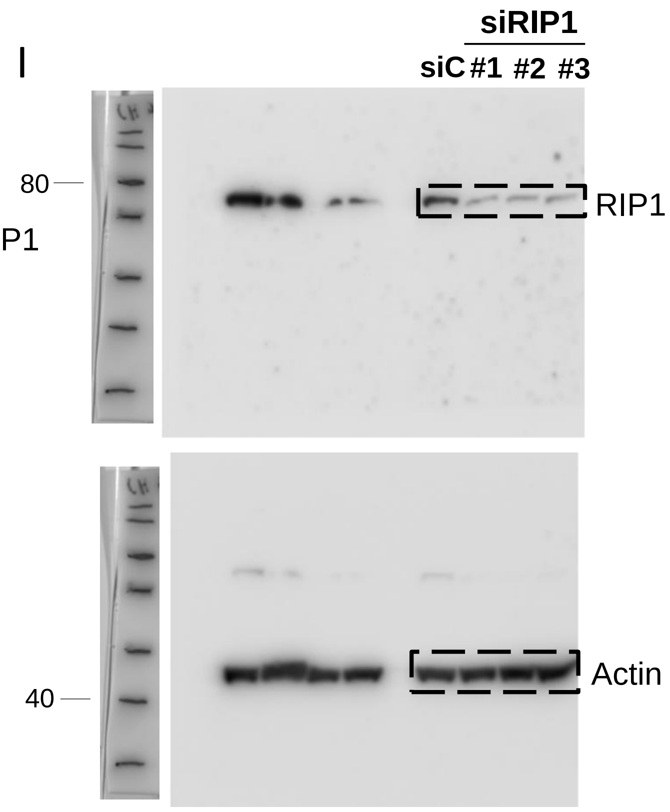

Bands in lanes outside the cropped area are from another experiment

Full-length blots from Figure 5

A

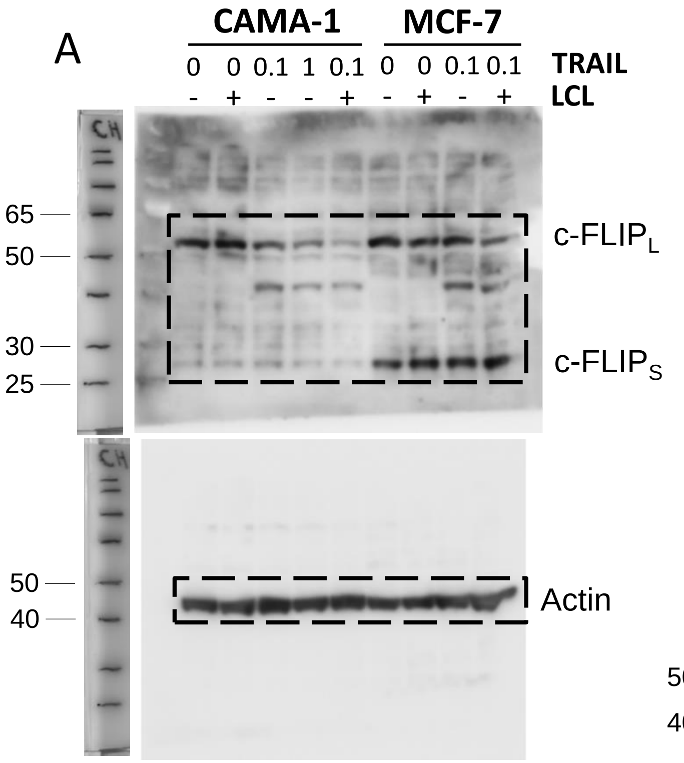

B

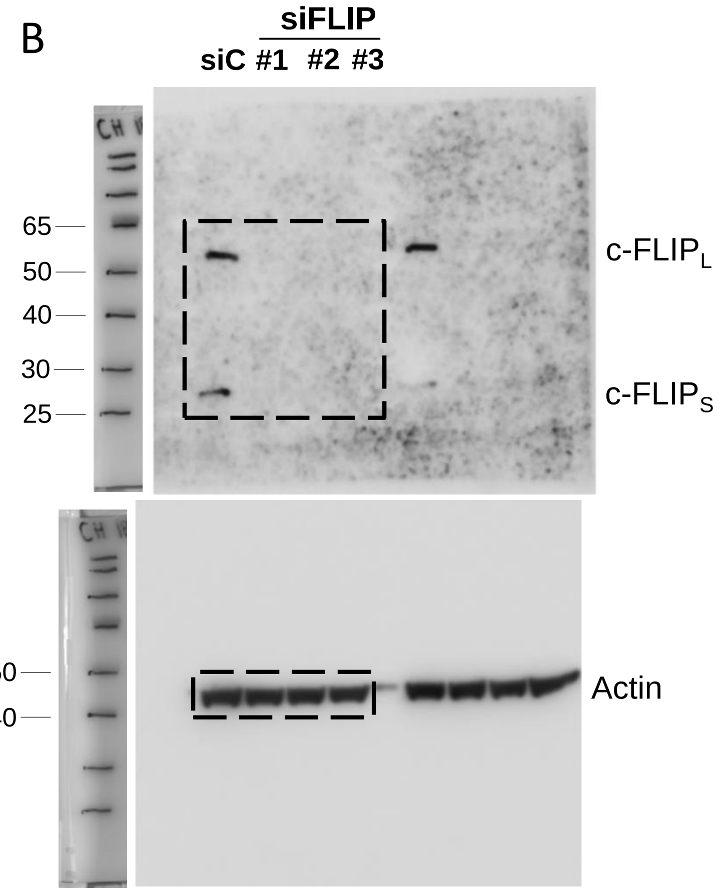

Bands in lanes outside the cropped area are from another experiment

D

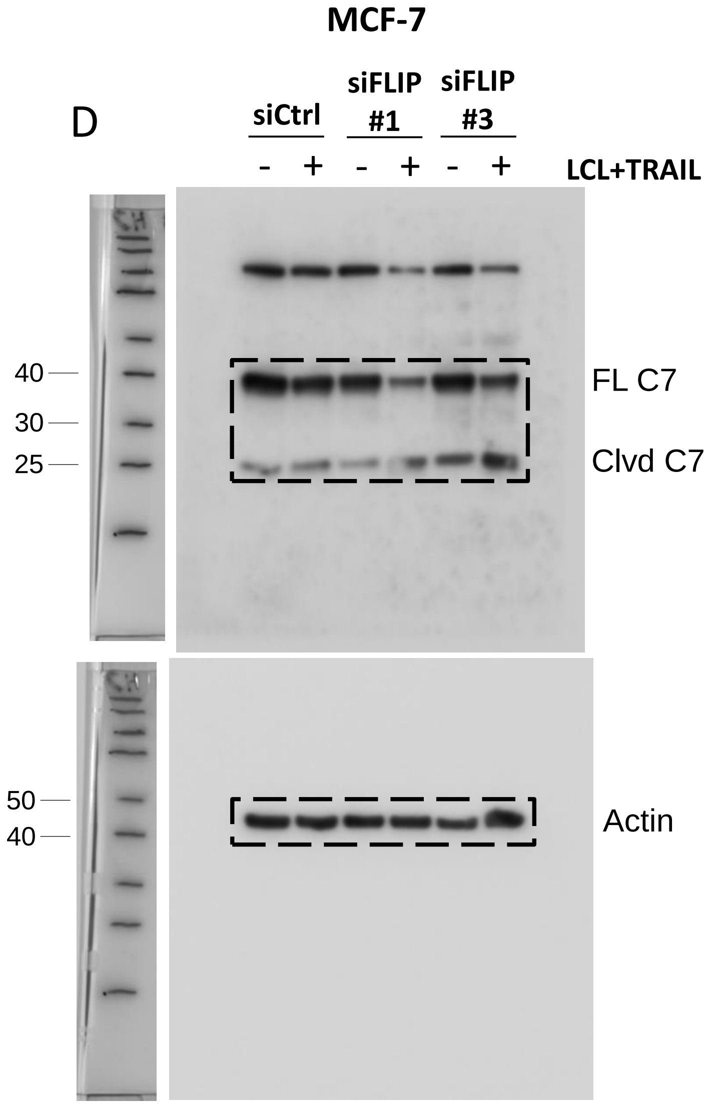

Full-length blots from Figure 5 continued

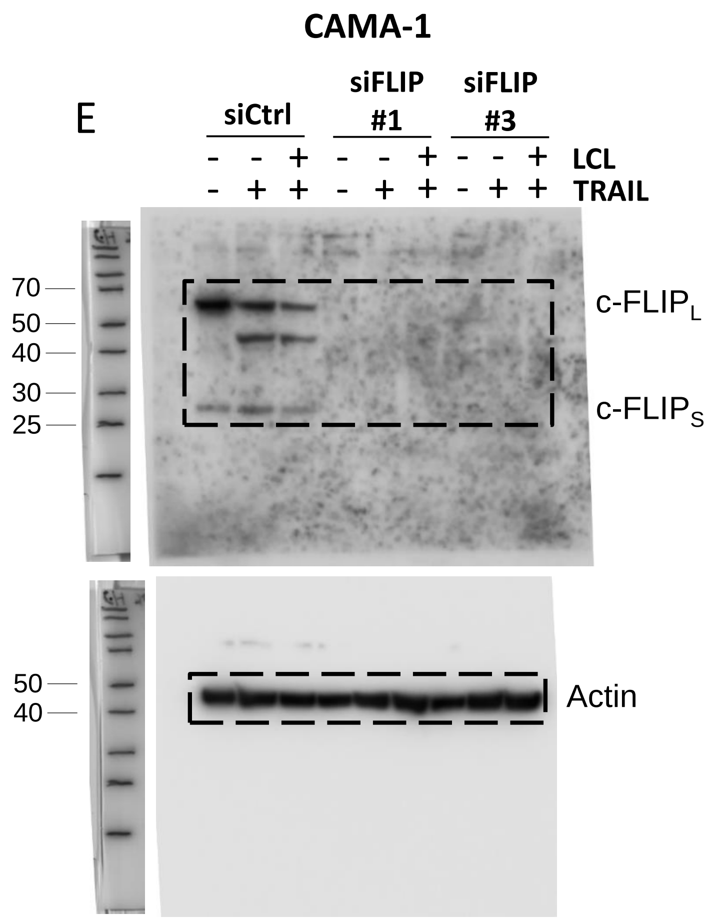

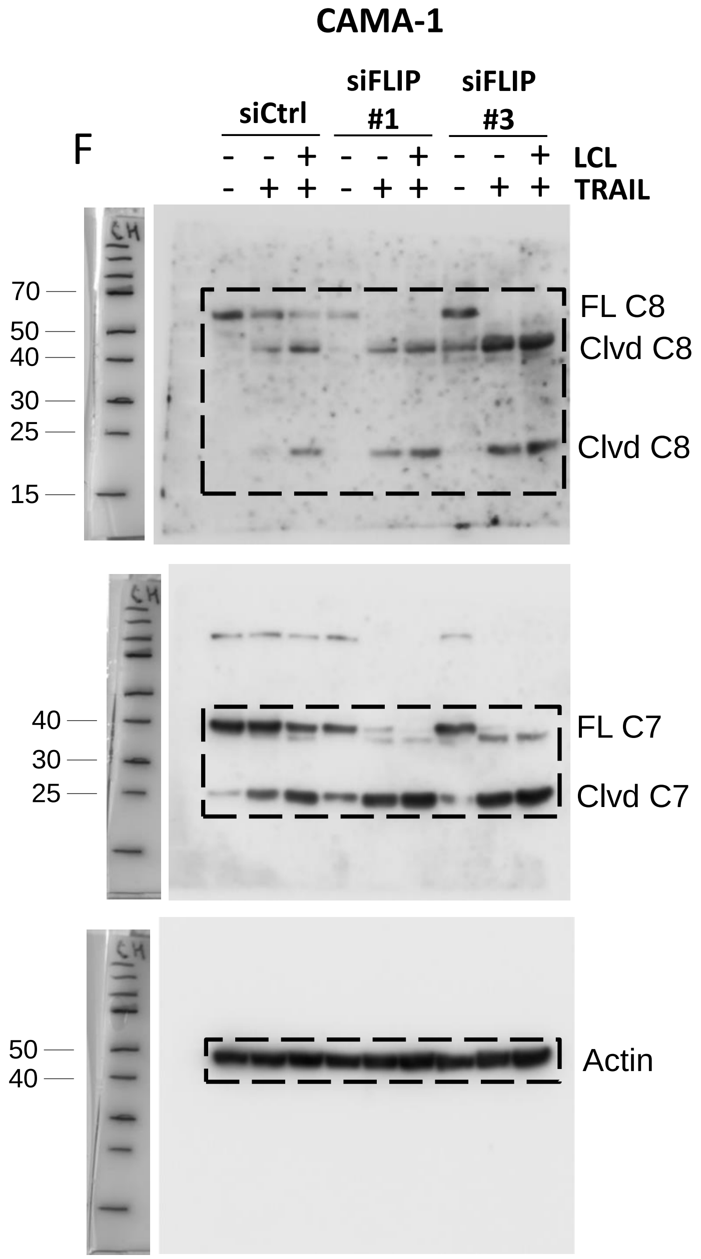

Full-length blots from Figure 5 continued

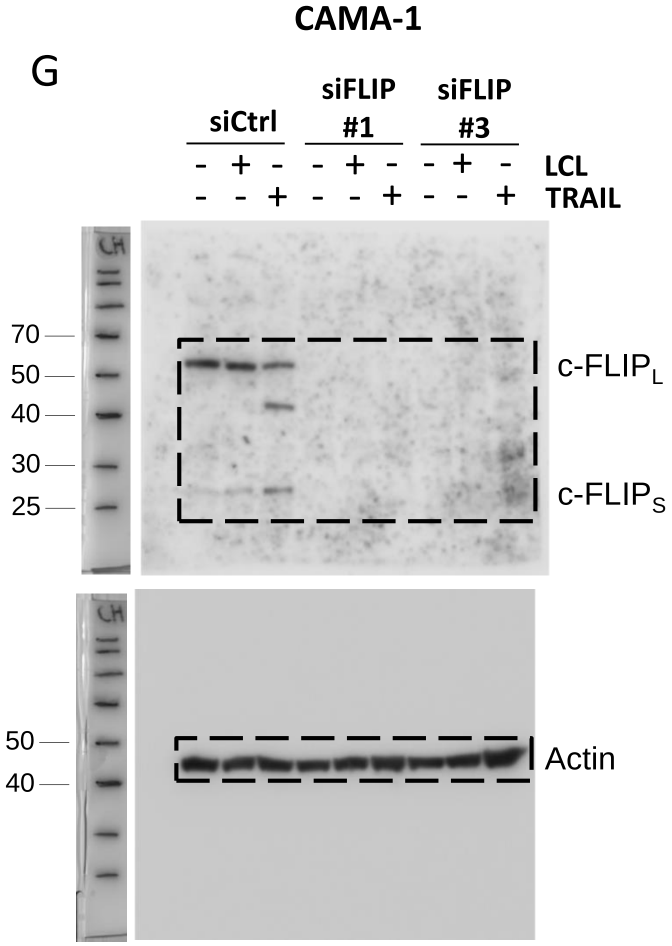

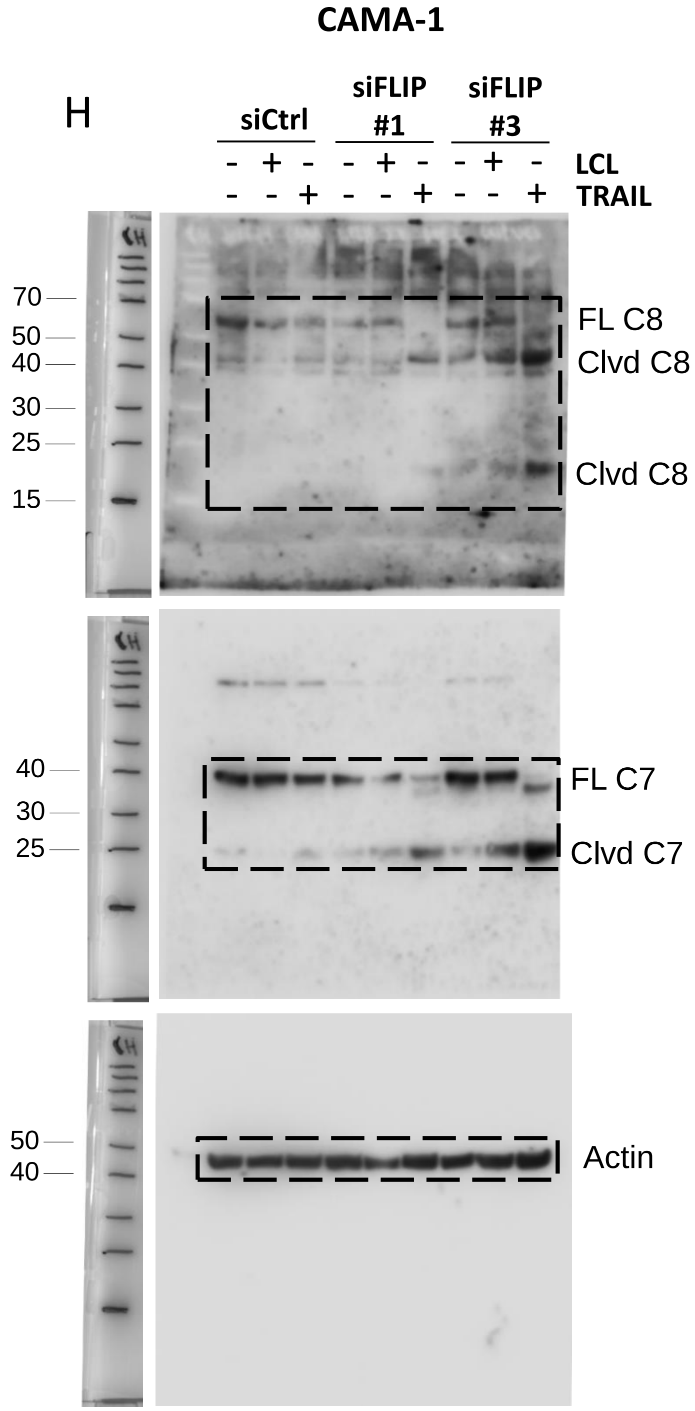

Full-length blots from Figure 6

A

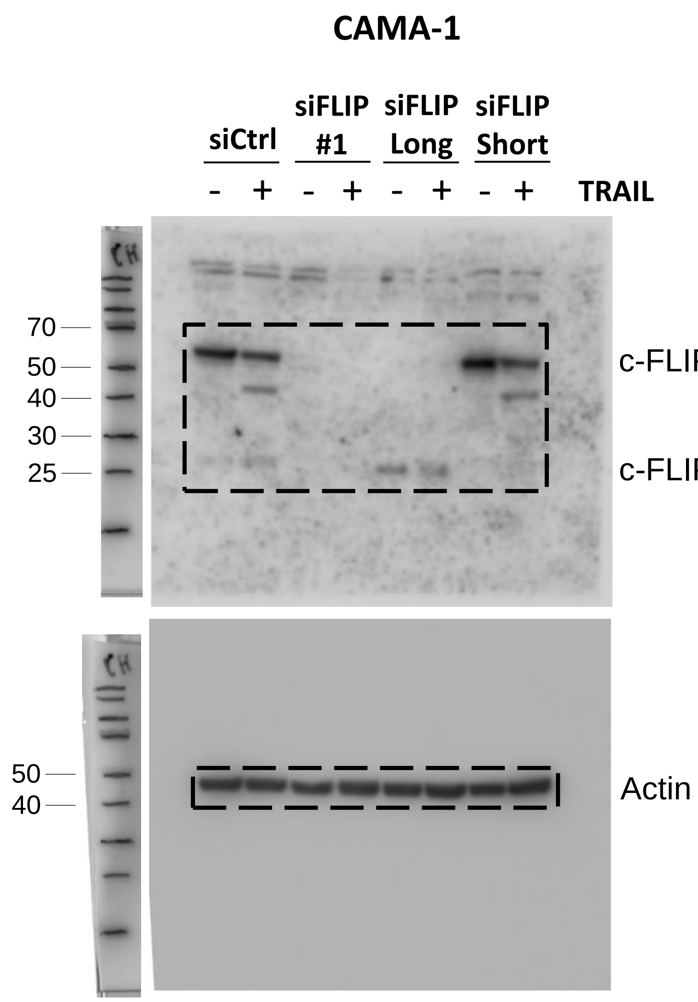

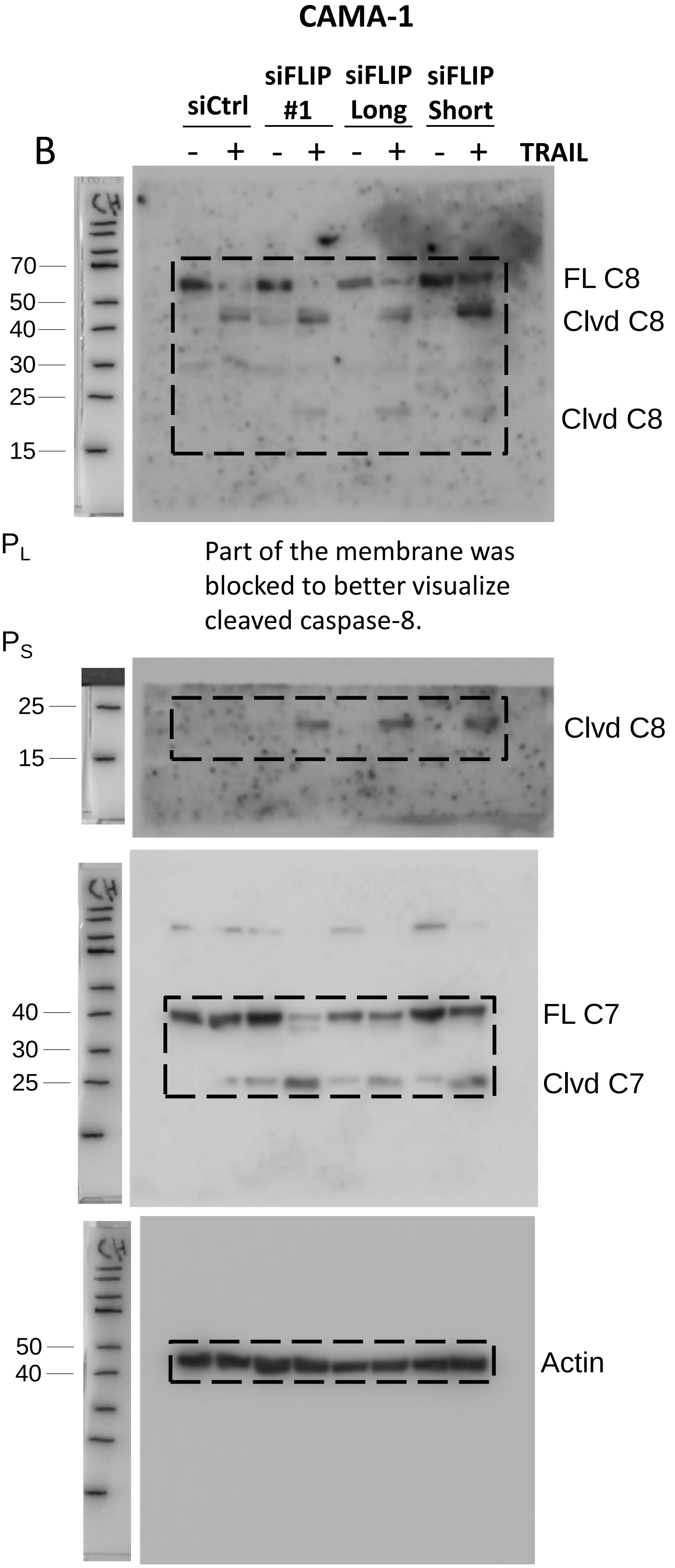

Full-length blots from Figure 6 continued

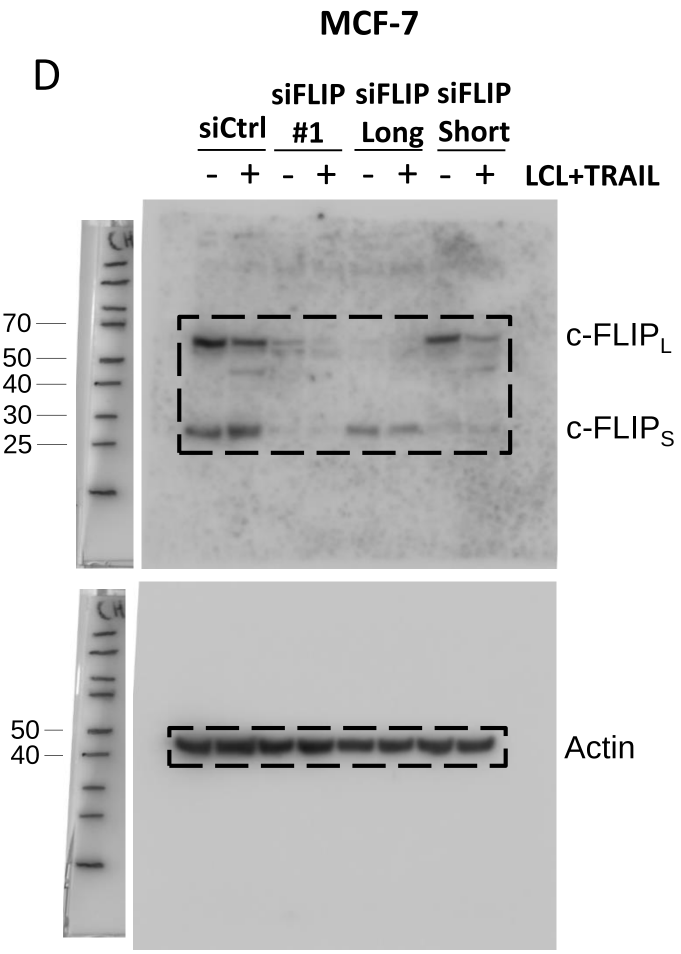

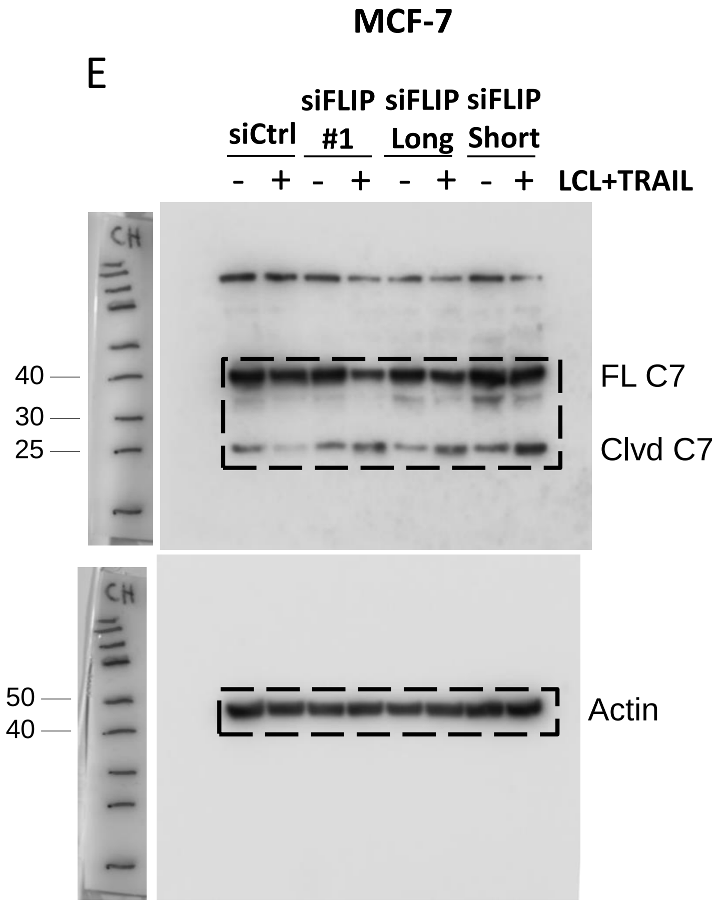

Supplement: Supplementary file 1 [file cimb-44-00327-s001.zip › Figure S3.pdf]
