# Supplementary figures and images for: Induction of Breast Cancer Cell Apoptosis by TRAIL and Smac Mimetics: Involvement of RIP1 and cFLIP
Source: Curr Issues Mol Biol. 2022 Oct 11;44(10):4803–21. doi: 10.3390/cimb44100327 (PMC9600666; doi:10.3390/cimb44100327)

MCF-7

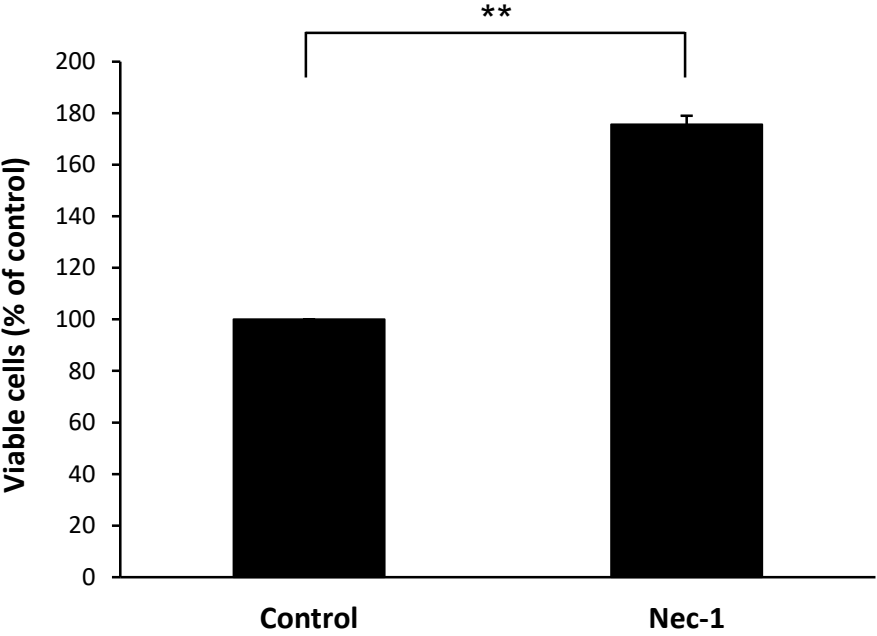

Supplement: Supplementary file 1 [file cimb-44-00327-s001.zip › Figure S1.pdf]

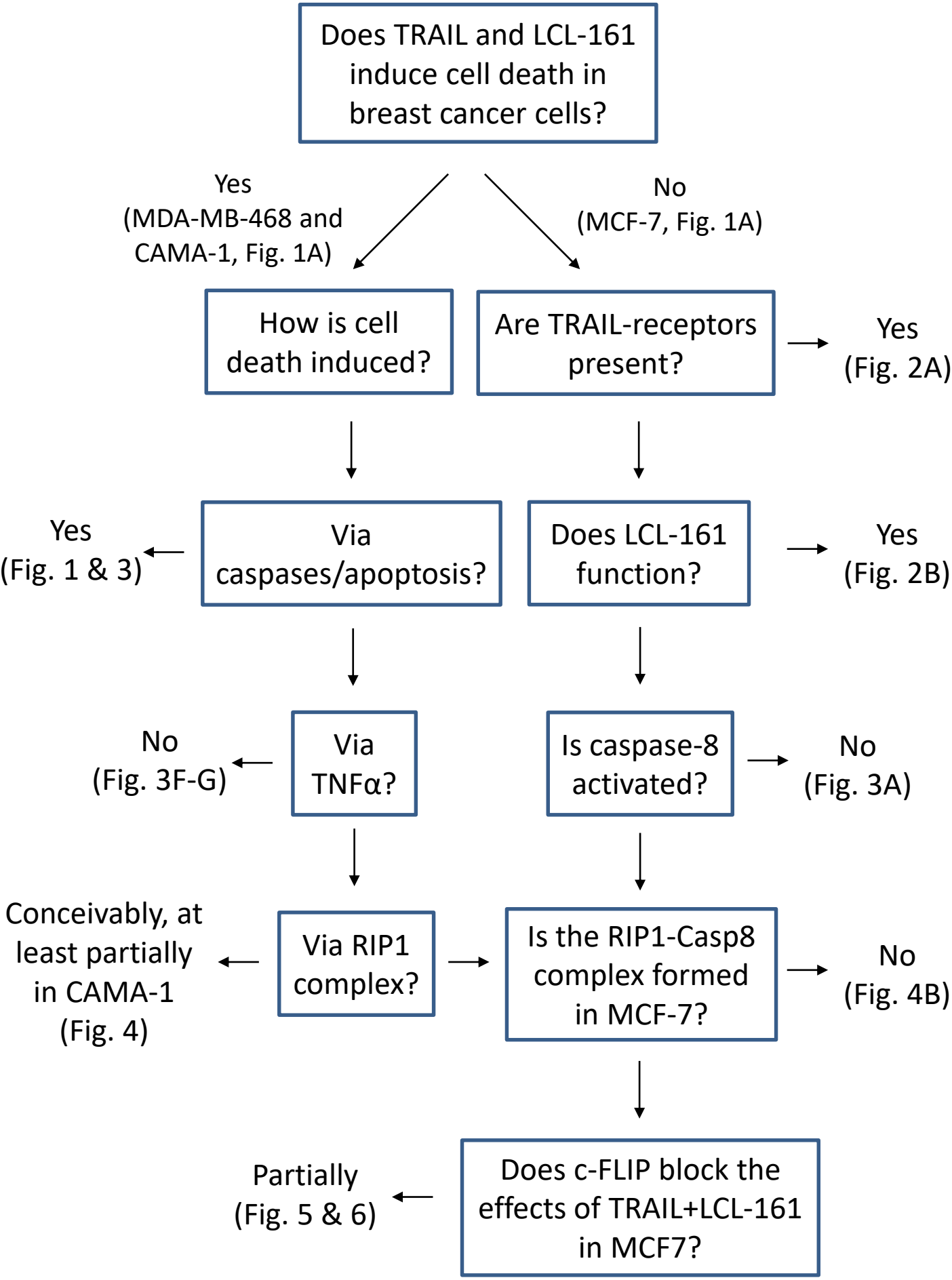

Supplement: Supplementary file 1 [file cimb-44-00327-s001.zip › Figure S2.pdf]
